# Copper-catalyzed Direct 2-Arylation of Benzoxazoles and Benzoimidazoles with Aryl Bromides and Cytotoxicity of Products

**DOI:** 10.1038/srep43758

**Published:** 2017-03-03

**Authors:** Nan-Nan Jia, Xin-Chuan Tian, Xiao-Xia Qu, Xing-Xiu Chen, Ya-Nan Cao, Yun-Xin Yao, Feng Gao, Xian-Li Zhou

**Affiliations:** 1 School of Life Science and Engineering, Southwest Jiaotong University, Chengdu 610031, P. R. China; 2 Department of Chinese Traditional Herbal, Agronomy College, Sichuan Agriculture University, Chengdu 611130, P. R. China

## Abstract

An efficient copper-catalyzed direct 2-arylation of benzoxazoles and benzoimidazoles with aryl bromides is presented. The CuI/PPh_3_-based catalyst promotes the installation of various aryl and heteroaryl groups through a C-H activation process in good to excellent yields. The cytotoxicity of obtained 2-aryl benzoxazoles (benzoimidazoles) was also evaluated and 1-methyl-2-(naphthalen-1-yl)benzoimidazole showed potential cytotoxicity.

2-Arylbenzoxazoles are important heterocyclic motif widely found in bioactive molecules^1^, pharmaceuticals^2^ and natural products^3^. 2-Substitued benzoxazoles are also fundamental scaffolds to construct novel ligands^4^,^5^,^6^,^7^ and materials^8^,^9^. As such, four main strategies for their preparation have been reported: (1) Transition metal-catalysed direct arylation of bezoxazoles with aryl halides^10^,^11^,^12^,^13^,^14^,^15^,^16^; (2) Intermolecular cyclization of 2-aminophenol with aldehydes^17^,^18^,^19^; (3) Intramolecular cyclization of halobenzanilides^20^,^21^; and (4) Ring-opening-coupling-recyclization of benzoxazoles with aromatic aldehydes or benzoyl chloride^22^,^23^. Among them, metal-catalysed direct arylation of C-H bond presents an economically attractive strategy to afford diverse 2-arylbenzoxazoles^24^,^25^,^26^,^27^,^28^. Convenient electrophiles, especially aryl halogens (ArX, X = Cl, Br, I), have been employed as the most widely used arylating reagents owing to their commercial availability and substituted diversity^29^,^30^,^31^,^32^.

Cross-coupling of benzoxazoles with aryl bromides catalysed by Palladium/Ligand system is an efficient protocol for the preparation of 2-arylbenzoxazoles^33^,^34^. Besides that, aryl chlorides are also an alternative coupling partner under Pd/N-heterocyclic carbene catalytic system (Fig. 1A)^35^. Except for noble metal Pd, cheap copper was found as qualified catalysis in the direct arylation of benzoxazoles with aryl iodides (Fig. 1B)^11^,^12^,^36^,^37^,^38^,^39^. Although efficiency, the synthesis of 2-arylbenzoxazoles *via* transition metal-catalysed direct arylation reaction with aryl halides suffered from the usage of noble metal or/and expensive aryl iodide. Recently, two copper-catalyzed direct arylation processes of benzoxazoles with aryl bromides were reported. But the employment of complex ligand^13^ or pre-preparation of nano-copper catalysis^14^ limited their widely application. Previously, we reported a room-temperature Pd/Nixantphos catalysed direct 2-arylation of benzoxazoles with aryl bromides^34^. In our continuous investigation on the metal catalysed synthesis 2-arylbenoxazoles, we report a Cu/PPh_3_-catalyzed direct 2-arylation of benzoxazoles with aryl bromides (Fig. 1C). At the same time, the cytotoxicity of afforded products is also tested.

## Results

We initiated the direct 2-aryation by testing six bases (LiO*t*Bu, NaO*t*Bu, KO*t*Bu, Na_2_CO_3_, K_2_CO_3_, NaOH) in DMF at 135 °C for 8 hours under the CuI/PPh_3_ (10 mol%/20 mol%) catalytic system, using benzoxazole (**1a**, 1.0 equiv.) and 1-bromo-4-*tert*-butylbenzene (**2a**, 1.2 equiv., Table 1, entries 1–6). Interestingly, all the bases can promote the direct arylation to furnish corresponding coupling products 2-(4-(*tert*-butyl)phenyl)benzoxazole (**3a**), with K_2_CO_3_ affording the desired compound in 88% yield after 8 hours. Meanwhile, weaker base showed more efficiency than strong base, which suggested the pKa of 2-H is reduced *via* the chelation of Cu ion with Nitrogen atom of benzoxazole. When the ligand was changed from PPh_3_ to Phen (1,10-phenanthroline), Xantphos, or Nixantphos, the yields dropped dramatically (Table 1, entries 7–9). Screening of different copper sources in the arylation reaction indicated that only CuCl gave arylated product in 10% yield and Cu(II) almost cannot catalyse this coupling (Table 1, entries 10–12). Considering the Cu(I) need 3equiv. of PPh_3_ to form stable Cu(PPh_3_)_3_I complex, the loading of CuI/PPh_3_ was changed to 5 mol%/15 mol% and the ratio of **1a**:**2a** was changed to 1.2:1, the coupling product **3a** was afforded in 58% yield and unconverted benzoxazole **1a** was detected (Table 1, Entry 13). Prolonging the reaction time to 24 h, the desired product **3a** was isolated in 95% yield (Table 1, Entry 14). Finally, we found the combination of CuI (5 mol%), PPh_3_ (15 mol%), and K_2_CO_3_ (2.0 equiv) in DMF at 135 °C under nitrogen for 24 h as the best conditions for the direct arylation.

**Figure 2:**
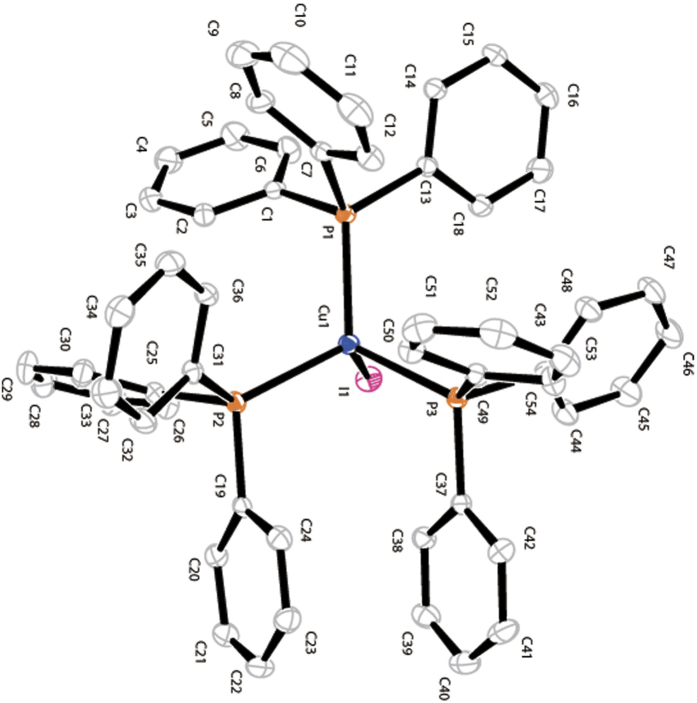
X-ray Structure of Cu(PPh_3_)_3_I (**4**).

With the best conditions in hand, we endeavoured to prepare air-stable Cu(PPh_3_)_3_I complex (**4**) in gram scales firstly and apply them in the catalytic arylation reaction in order to simplify the operation process. Cu(PPh_3_)_3_I complex (**4**) crystals were easy afforded *via* reaction of CuI (1.0 equiv.) with PPh_3_ (3.0 equiv.) in anhydrous DMF at 45° C for 3 h. The single crystal of Cu(PPh_3_)_3_I complex (**4**) was resolved by X-ray diffraction for the first time (Fig. 2, CCDC 1497357). Unfortunately, stable Cu(PPh_3_)_3_I complex (**4**) crystal showed weaker catalytic efficiency than newly prepared catalysis solution under the best conditions and afforded **3a** in 83% yield.

Based on the optimized arylation conditions (Table 1, entry 14), we examined a variety of aryl bromides in the cross-coupling process using newly prepared CuI/ PPh_3_ solution as catalysis (Table 2). In general, the direct arylation of benzoxazole (**1a**) exhibited good yields with a range of aryl bromides. Electron neutral 1-bromo-4-*tert*-butylbenzene, the parent bromobenzene, and 2-bromonaphthalene furnished the desired products in 95, 92 and 98% yield, respectively. Aryl bromides bearing electron-donating methoxyl group led to the 2-(6-methoxynaphthalen-2-yl)benzoxazole (**3d**) in 88% yield. Electron withdrawing substituents (4-F, 4-Cl, 4-CF_3_, 3-CF_3_ and 3-CN) on the aryl bromide were well tolerated, providing products in 78–94% yields. It is worth mentioning that pyridine bromides, such as 3-bromopyridine, 3-bromo-5-methylpyridine, 2-bromopyridine, 4-bromopyridine and 2-bromoquinoline, were excellent arylated reagents with benzoxazole to afford corresponding 2- pyridylbenzoxazoles (**3j**–**m**) in 82–92% yields. Moreover, 2-bromoquinoline, 3-bromoquinoline and 4-bromoquinoline were also excellent coupling partners with benzoxazole to give bioactive biheterocyclic products (**3n**–**p**) in 90%, 84%, and 82% yields. Sadly, furan and thiophene compounds cannot survive under the conditions owing to the high reaction temperature.

Having demonstrated the broad scope of the aryl bromides in the arylation reaction, we then briefly explored the coupling of 5-methoxybenzoxazole (**1b**) and 1-methyl-1*H*-benzoimidazole (**1c**) with electron-withdrawing group substituted 1-bromo-4-chlorobenzene (**2f**), electron neutral 2-bromonaphthalene (**2c**), electron- donating group substituted 2-bromo-6-methoxynaphthalene (**2d**) and sterically hindered 1-bromonaphthalene (**2m**). To our delight, corresponding arylated products can be obtained in moderate to good yields (Table 3). We also tried the direct arylation of benzothiazole with aryl bromides under our optimized conditions but failed.

Benzo-azole derivatives are a class of important heterocyclic compounds possessing remarkable and various biological activity^1^,^2^. Therefore, the *in vitro* cytotoxicity of obtained 2-aryl benzoxazoles and benzoimidazoles analogues against several human cancer cell lines was evaluated by classic MTT methods using paclitaxel as positive control (Table 4). Interestingly, all the 2-arylbenzoxazoles exhibited totally inactivity except for quinolinylbenzoxazoles (**3n–p**) showing weak cytotoxicity against BGC-823. In contrast, most of 2-arylbenzoimidazoles (**3t–v**) displayed potential cytotoxicity against BGC-823 but inactivity of 2-(4-chlorophenyl)-1-methyl-1*H*-benzoimidazole (**3s**). Comprehensive analysing the structure-activity relationship (SAR) of 2-aryl benzoxazoles and benzoimidazoles, we can draw the conclusion that: 1) Nitrogen atom is the essential to keep the cytotoxicity. 2) Big substituted groups at C-2 position contribute to the increasing of cytotoxicity. Based on the preliminary SAR results, we will design, synthesis and biological evaluation more N-alkyl-2-heterocycilic aryl benzoimidazoles analogues in our continuous research.

## Discussion

In summary, we have demonstrated a copper-catalysed direct 2-arylation of benzoxazoles and benzoimidazoles with aryl bromides. The cheap copper catalysis and PPh_3_ ligand promoted the cross-coupling reaction in good to excellent yields with broad substrate scope, which provides a promising method for the synthesis of pharmacologically significant 2-aryl benzoxazoles and benzoimidazoles derivatives. Meanwhile, preliminary cytotoxicity and SAR results of afforded products will give important guideline in the medicinal chemistry community of azoles.

## Methods

### General Information

All reactions were conducted under an inert atmosphere of dry argon. All reagents were purchased from TCI and used without further purification. N,N-Dimethylformamide (DMF), toluene and xylene were dried through activated 4 Å Molecular Sieves under argon. 1,4-dioxane was dried through calcium hydride and tetrahydrofuran (THF) with sodium. Solvents were commercially available and used as received without further purification. Reactions were monitored by thin layer chromatography (TLC) on silica gel plates (GF 254) using UV light to visualize the course of the reactions. NMR spectra were obtained using a Brüker 400 MHz Fourier-transform NMR spectrometer. High resolution mass spectrometry (HRMS) data were obtained on a Waters LC-TOF mass spectrometer (model LCT-XE Premier) using chemical ionization (CI) or electrospray ionization (ESI) in positive or negative mode, depending on the analyze. Chemical shifts (*δ*) are reported in ppm with TMS as internal standard. Abbreviations for signal couplings are: s, singlet; d, doublet; t, triplet; m, multiplet.

### General Procedure for the Cu-catalyzed arylation of benzoxazoles and benzoimidazoles

An oven-dried 10 mL reaction vial equipped with a stir bar was charged with benzoxazole (0.5 mmol) and K_2_CO_3_ (138.0 mg, 1.0 mmol, 2 equiv), and then sealed with a rubber stopper under an argon atmosphere. A solution (from a stock solution) of CuI (4.76 mg, 0.025 mmol) and PPh_3_ (19.7 mg, 0.075 mmol) in 1 mL of dry DMF was taken up by syringe and added to the reaction vial. Preparation of the stock solution from CuI (23.8 mg, 0.125 mmol) and PPh_3_ (98.4 mg, 0.375 mmol) in 5 mL of dry DMF was stirred for 1 h at 135 °C under argon. Aryl bromide (0.6 mmol, 1.2 equiv) was added to the reaction mixture by syringe. Note that solid aryl bromides were added to the reaction vial prior to addition of K_2_CO_3_. The reaction mixture was stirred for 24 h at 135 °C, quenched with two drops of H_2_O, diluted with 3 mL of ethyl acetate, and filtered over a pad of Na_2_SO_4_ and silica. The pad was rinsed with additional ethyl acetate, and the solution was concentrated *in vacuo*. The crude material was loaded onto a silica gel column and purified by flash chromatography.

### Determination of cell viability by MTT assay

Cells were plated in the RPMI 1640 with 10% fetal calf serum media on 96-well plates in a total volume of 100 μL with a density of 1 × 10^4^ cells mL^−1^. Triplicate wells were treated with media and tested compounds. The plates were incubated at 37 °C in 5% CO_2_ for 72 h. Cell viability was determined based on mitochondrial conversion of 3[4,5-dimethylthiazol-2-yl]-2,5-diphenyltetrazolium bromide (MTT, Sigma) to formazan. The amount of MTT converted to formazan is a sign of the number of viable cells. Each well was supplemented with 50 mL of a 1 mg mL^−1^solution of MTT in uncompleted media. The plates were incubated in 37 °C, 5% CO_2_ for an additional 4 h. The media was carefully removed from each well and then 200 μL of DMSO was added. The plates were gently agitated until the reaction color was uniform and the OD_570_ was determined using a microplate reader (Wellscan MK3, Labsystems Dragon). Microsoft^®^ Excel 2010 was used to analyze data. Media-only treated cells served as the indicator of 100% cell viability. The 50% inhibitory concentration (IC_50_) was defined as the concentration that reduced the absorbance of the untreated wells by 50% of the control in the MTT assay.

### 2-(4-tert-Butylphenyl)benzoxazole (3a)

White solid (119 mg, 95% yield); ^1^H NMR (400 MHz, CDCl_3_): δ = 8.22 (d, *J* = 8.5 Hz, 2H), 7.81–7.79 (m, 1H), 7.62–7.57 (m, 3H), 7.38–7.36 (m, 2H), 1.40 (s, 9H) ppm; ^13^C NMR (100 MHz, CDCl_3_): δ = 163.3, 155.1, 150.7, 142.2, 127.5, 125.9, 124.9, 124.5, 124.4, 119.9, 110.5, 35.1, 31.2 ppm. The ^1^H and ^13^C NMR data for this compound match the literature data^34^.

### 2-Phenylbenzoxazole (3b)

White solid (89 mg, 92% yield); ^1^H NMR (400 MHz, CDCl_3_): δ = 8.30–8.28 (m, 2H), 7.82–7.80 (m, 1H), 7.63–7.60 (m, 1H), 7.57–7.55 (m, 3H), 7.40–7.37 (m, 2H) ppm; ^13^C NMR (100 MHz, CDCl_3_): δ = 163.1, 150.8, 142.1, 131.5, 128.9, 127.6, 127.2, 125.1, 124.6, 120.0, 110.6 ppm. The ^1^H and ^13^C NMR data for this compound match the literature data^34^.

### 2-(Naphthalen-2-yl)benzoxazole (3c)

Yellow solid (120 mg, 98% yield); ^1^H NMR (400 MHz, CDCl_3_): δ = 8.80 (s, 1H), 8.34 (dd, *J* = 8.6 Hz, 1.7 Hz, 1H), 8.02–7.98 (m, 2H), 7.92–7.90 (m, 1H), 7.85–7.83 (m, 1H), 7.66–7.58 (m, 3H), 7.41–7.39 (m, 2H) ppm; ^13^C NMR (100 MHz, CDCl_3_): δ = 163.2, 150.9, 142.2, 134.8, 133.0, 129.0, 128.8, 128.2, 127.9, 127.8, 126.9, 125.2, 124.7, 124.4, 124.0, 120.1, 110.6 ppm. The ^1^H and ^13^C NMR data for this compound match the literature data^34^.

### 2-(6-Methoxynaphthalen-2-yl)benzoxazole (3d)

Yellow solid (121 mg, 88% yield); ^1^H NMR (400 MHz, CDCl_3_): δ = 8.68 (s, 1H), 8.27 (dd, *J* = 8.6 Hz, 1.6 Hz, 1H), 7.88–7.80 (m, 3H), 7.62–7.60 (m, 1H), 7.38–7.36 (m, 2H), 7.22 (dd, *J* = 8.9 Hz, 2.5 Hz, 1H), 7.16 (s, 1H), 3.94 (s, 3H) ppm; ^13^C NMR (100 MHz, CDCl_3_): δ = 163.5, 159.2, 150.8, 142.3, 136.3, 130.5, 128.4, 128.0, 127.5, 124.9, 124.6, 124.6,122.2, 119.8, 119.8, 110.5, 105.9, 55.4 ppm. HRMS calculated for C_18_H_14_NO_2_, 276.1025, found 276.1038, [M+H]^+^.

### 2-(4-Fluorophenyl)benzoxazole (3e)

Yellow solid (93.7 mg, 88% yields); ^1^H NMR (400 MHz, CDCl_3_): δ = 8.30–8.27 (m, 2H), 7.80–7.78 (m, 1H), 7.61–7.59(m, 1H), 7.39–7.37 (m, 2H), 7.26–7.22 (m, 2H) ppm; ^13^C NMR (100 MHz, CDCl_3_): δ = 164.8 (d, *J* = 252.8 Hz), 162.2, 150.8, 142.1, 129.9 (d, *J* = 8.9 Hz), 125.2, 124.7, 123.5, 120.0, 116.2 (d, *J* = 22.2 Hz), 110.6 ppm. The ^1^H and ^13^C NMR data for this compound match the literature data^34^.

### 2-(4-Chlorophenyl)benzoxazole (3f)

White solid (99.6 mg, 87% yield); ^1^H NMR (400 MHz, CDCl_3_): δ = 8.19 (d, *J* = 8.7 Hz, 2H), 7.80–7.78 (m, 1H), 7.60–7.58 (m, 1H), 7.52–7.50 (d, *J* = 8.7 Hz, 2H), 7.39–7.37 (m, 2H) ppm; ^13^C NMR (100 MHz, CDCl_3_): δ = 162.1, 150.8, 142.0, 137.8, 129.3, 128.9, 125.7, 125.4, 124.87, 120.1, 110.6 ppm. The ^1^H and ^13^C NMR data for this compound match the literature data^34^.

### 2-(4-(Trifluoromethyl)phenyl)benzoxazole(3g)

White solid. (111.7 mg, 85% yield); ^1^H NMR (400 MHz, CDCl_3_): δ = 8.39 (d, *J* = 8.1 Hz, 2H), 7.84–7.80 (m, 3H), 7.65–7.62 (m, 1H), 7.43–7.41 (m, 2H) ppm; ^13^C NMR (100 MHz, CDCl_3_): δ = 161.5, 150.9, 141.9, 133.0 (m, *J* = 32.8 Hz), 130.5, 127.9, 125.9 (m, *J* = 3.8 Hz), 125.8, 125.0, 122.4, 120.4, 110.8 ppm. The ^1^H and ^13^C NMR data for this compound match the literature data^36^.

### 2-(3-(trifluoromethyl)phenyl)benzoxazole (3h)

White solid (102.5 mg, 78% yield); ^1^H NMR (400 MHz, CDCl_3_): δ = 8.55 (s, 1H), 8.44 (d, *J* = 7.8 Hz, 1H), 7.83–7.80 (m, 2H), 7.70–7.62 (m, 2H), 7.42–7.40 (m, 2H) ppm; ^13^C NMR (100 MHz, CDCl_3_): δ = 161.5, 150.8, 141.9, 131.6 (m, *J* = 32.7 Hz), 130.6, 129.5, 128.0, 127.9 (dd, *J* = 7.3 Hz, 3.6 Hz), 125.7, 124.9, 124.5 (m, *J* = 3.8 Hz), 122.4, 120.3, 110.8 ppm. The ^1^H and ^13^C NMR data for this compound match the literature data^34^.

### 3-(benzoxazol-2-yl)benzonitrile (3i)

White solid (103.4 mg, 94% yield); ^1^H NMR (400 MHz, CDCl_3_): δ = 8.55 (s, 1H), 8.48 (d, *J* = 8.0 Hz, 1H), 7.83–7.81 (m, 2H), 7.69–7.65 (t, *J* = 8.0 Hz, 1H), 7.64–7.62 (m, 1H), 7.44–7.41 (m, 2H) ppm; ^13^C NMR (100 MHz, CDCl_3_): δ = 160.6, 150.8, 141.8, 134.4, 131.4, 131.0, 129.9, 128.6, 126.0, 125.1, 120.5, 117.9, 113.5, 110.9 ppm. The ^1^H and ^13^C NMR data for this compound match the literature data^40^.

### 2-(pyridin-3-yl)benzoxazole(3j)

White solid (84.2 mg, 86% yield); ^1^H NMR (400 MHz, CDCl_3_): δ = 9.46 (s, 1H), 8.75 (dd, *J* = 4.8 Hz, 1.5 Hz, 1H), 8.49 (d, *J* = 8.0 Hz, 1H), 7.79–7.77 (m, 1H), 7.60–7.58 (m, 1H), 7.46–7.44 (m, 1H), 7.38–7.36 (m, 2H) ppm; ^13^C NMR (100 MHz, CDCl_3_): δ = 160.6, 152.0, 150.7, 148.8, 141.8, 134.7, 125.7, 124.9, 123.7, 123.6, 120.3, 110.8 ppm. The ^1^H and ^13^C NMR data for this compound match the literature data^41^.

### 2-(5-methylpyridin-3-yl)benzoxazole (3k)

White solid (86.1 mg, 82% yield); m.p. = 110–112 °C; ^1^H NMR (400 MHz, CDCl_3_): δ = 9.28 (s, 1H), 8.60 (s, 1H), 8.34 (s, 1H), 7.80–7.79 (m, 1H), 7.63–7.60 (m, 1H), 7.40–7.38 (m, 2H), 2.46 (s, 3H) ppm; ^13^C NMR (100 MHz, CDCl_3_): δ = 161.0, 152.7, 150.7, 146.0, 141.8, 135.1, 133.5, 125.6, 124.9, 123.0, 120.2, 110.8, 18.4 ppm; IR (thin film): 3116, 1560, 1529, 1454, 1441, 1244, 1201, 1118, 1015, 819 cm^−1^; HRMS calculated for C_13_H_11_N_2_O, 211.0871, found 211.0882, [M+H]^+^ .

### 2-(pyridin-2-yl)benzoxazole (3l)

White solid (90.0 mg, 92% yield); ^1^H NMR (400 MHz, CDCl_3_): δ = 8.80 (s, 2H), 8.34 (d, *J* = 8.0 Hz, 1H), 7.87 (t, *J* = 8.0 Hz, 1H), 7.84–7.78 (m, 1H), 7.68–7.58 (m, 1H), 7.46–7.34 (m, 3H) ppm; ^13^C NMR (100 MHz, CDCl_3_): δ = 161.4, 151.0, 150.3, 146.0, 141.7, 137.0, 126.0, 125.5, 124.9, 123.4, 120.6, 111.2 ppm. The ^1^H and ^13^C NMR data for this compound match the literature data^10^.

### 2-(pyridin-4-yl)benzoxazole (3m)

Yellow solid (87.2 mg, 89% yield); ^1^H NMR (400 MHz, CDCl_3_): δ = 8.8 (s, 2H), 8.08 (d, *J* = 6.0 Hz, 1H), 7.85–7.79 (m, 1H), 7.65–7.58 (m, 1H), 7.48–7.36 (m, 2H) ppm; ^13^C NMR (100 MHz, CDCl_3_): δ = 160.6, 150.9, 150.7, 141.7, 134.3, 126.3, 125.1, 121.0, 120.7, 110.9 ppm. The ^1^H and ^13^C NMR data for this compound match the literature data^10^.

### 2-(isoquinolin-3-yl)benzoxazole (3n)

Yellow solid (110.7 mg, 90% yield); ^1^H NMR (400 MHz, CDCl3): δ = 8.48 (d, *J* = 8.4 Hz, 1H), 8.36(d, *J* = 8.4 Hz, 2H), 7.89 (t, *J* = 8.4 Hz, 2H), 7.82 (dt, *J*_1_ = 8.4 Hz, *J*_2_ = 1.6 Hz, 1H), 7.78–7.71 (m, 1H), 7.68–7.62 (m, 1H), 7.50–7.41 (m, 2H) ppm; ^13^C NMR (101 MHz, CDCl_3_) δ = 161.6, 151.3, 148.1, 145.9, 141.8, 137.3, 130.4, 130.3, 128.7, 128.1, 127.7, 126.3, 125.0, 120.8, 120.3, 111.5 ppm. The ^1^H and ^13^C NMR data for this compound match the literature data^10^.

### 2-(Quinolin-4-yl)benzoxazole (3o)

Yellow solid (103.3 mg, 84% yield); ^1^H NMR (400 MHz, CDCl3): δ = 9.46 (d, *J* = 6.8 Hz, 1H), 9.43 (s, 1H), 9.36 (s, 1H), 8.06 (d, *J* = 6.8 Hz, 1H), 7.91 (t, *J* = 6.8 Hz, 1H), 7.89–7.85 (m, 1H), 7.71 (t, *J* = 6.8 Hz, 1H), 7.69–7.63 (m, 1H), 7.46–7.37 (m, 2H) ppm; ^13^C NMR (101 MHz, CDCl_3_) δ = 161.0, 155.6, 150.1, 145.2, 142.0, 132.8, 132.2, 128.4, 128.3, 127.9, 125.7, 125.6, 124.7, 120.3, 117.8 ppm. The ^1^H and ^13^C NMR data for this compound match the literature data^34^.

### 3-Benzooxazol-2-ylquinoline (3p)

Yellow solid (100.8 mg, 82% yield); ^1^H NMR (400 MHz, CDCl_3_): δ = 9.75 (s, 1H), 9.00 (s, 1H), 8.20 (d, *J* = 8.5 Hz, 1H), 7.98 (d, *J* = 7.9 Hz, 1H), 7.86–7.81 (m, 2H), 7.67–7.63 (m, 2H), 7.43–7.41 (m, 2H) ppm; ^13^C NMR (100 MHz, CDCl_3_): δ = 161.0, 150.8, 149.1, 148.6, 141.9, 135.3, 131.3, 129.6, 128.7, 127.7, 127.2, 125.7, 125.0, 120.4, 120.3, 110.8 ppm. The ^1^H and ^13^C NMR data for this compound match the literature data^42^.

### 2-(4-Chlorophenyl)-5-methoxybenzoxazole (3q)

Yellow solid (108.7 mg, 84% yield); m.p. = 120–121 °C; ^1^H NMR (400 MHz, CDCl_3_): δ = 8.18 (d, *J* = 8.7 Hz, 2H), 7.53–7.46 (m, 3H), 7.27 (d, *J* = 2.5 Hz, 1H), 7.00–6.97 (m, 1H), 3.90 (s, 3H) ppm; ^13^C NMR (100 MHz, CDCl_3_): δ = 162.8, 157.5, 145.4, 142.8, 137.7, 129.3, 128.7, 125.8, 114.0, 110.8, 102.9, 56.0 ppm. IR (thin film): 1626, 1567, 1468, 1322, 1234, 1155, 998, 816 cm^−1^; HRMS calculated for C_14_H_11_ClNO_2_, 260.0478, found 260.0482, [M+H]^+^.

### 5-Methoxy-2-(naphthalen-2-yl)benzoxazole (3r)

Yellow solid (121 mg, 88% yield); m.p. = 136–137 °C; ^1^H NMR (400 MHz, CDCl_3_): δ = 8.78 (s, 1H), 8.32 (dd, *J* = 8.6 Hz, 1.6 Hz, 1H), 8.02–7.91(m, 3H), 7.63–7.57 (m, 2H), 7.52 (d, *J* = 8.9 Hz.1H).7.32 (d, *J* = 2.5 Hz, 1H), 7.00 (dd, *J* = 8.9 Hz, 2.5 Hz, 1H), 3.92 (s, 3H) ppm; ^13^C NMR (100 MHz, CDCl_3_): δ = 164.0, 157.5, 145.6, 143.1, 134.7, 133.0, 128.9 (d, *J* = 18.3 Hz), 128.0–127.7 (m), 126.9, 124.5, 123.9, 113.8, 110.7, 102.9, 56.0 ppm. IR (thin film): 3081, 3005, 1565, 1502, 1437, 1409, 1325, 1211, 1006, 867 cm^−1^; HRMS calculated for C_18_H_14_NO_2_, 276.1025, found 276.1029, [M+H]^+^.

### 2-(4-Chlorophenyl)-1-methyl-1H-benzoimidazole(3s)

White solid (98 mg, 81% yield) as a. ^1^H NMR (400 MHz, CDCl_3_): δ = 7.85–7.83 (m, 1H), 7.73 (d, *J* = 8.6 Hz, 2H), 7.52 (d, *J* = 8.6 Hz, 2H), 7.41–7.39 (m, 1H), 7.36–7.33 (m, 2H), 3.86 (s, 3H) ppm; ^13^C NMR (100 MHz, CDCl_3_): δ = 152.6, 142.9, 136.6, 136.0, 130.7, 129.0, 128.7, 123.1, 122.7, 119.9, 109.7, 31.7 ppm. The ^1^H and ^13^C NMR data for this compound match the literature data^43^.

### 1-Methyl-2-(naphthalen-2-yl)-1H-benzoimidazole (3t)

Yellow solid (109.6 mg, 85% yield); m.p. = 129–130 °C; ^1^H NMR (400 MHz, CDCl_3_): δ = 8.28 (s, 1H), 8.02 (d, *J* = 8.5 Hz, 1H), 7.98–7.90 (m, 4H), 7.61–7.58 (m, 2H), 7.46–7.44 (m, 1H), 7.38–7.36 (m, 2H), 3.95(s, 3H) ppm; ^13^C NMR (100 MHz, CDCl_3_): δ = 153.8, 143.1, 136.7, 133.6, 133.0, 129.4, 128.6, 128.5, 127.9, 127.6, 127.3, 126.8, 126.4, 122.9, 122.6, 119.9, 109.7, 31.9 ppm. IR (thin film): 1738, 1567, 1133, 1006, 748 cm^-1^; HRMS calculated for C_18_H_15_N_2_, 259.1235, found 259.1239, [M+H]^+^.

### 2-(6-Methoxynaphthalen-2-yl)-1-methyl-1H-benzoimidazole (3u)

Yellow solid (118.0 mg, 82% yield); m.p. = 131–132 °C; ^1^H NMR (400 MHz, CDCl_3_): δ = 8.20 (s, 1H), 7.91–7.85 (m, 4H), 7.44–7.42 (m, 1H), 7.37–7.34 (m, 2H), 7.26–7.22 (m, 2H), 3.98 (s, 3H), 3.94 (s, 3H) ppm; ^13^C NMR (100 MHz, CDCl_3_): δ = 158.7, 154.0, 143.1, 136.7, 135.1, 130.1, 129.2, 128.5, 127.3, 126.9, 125.3, 122.7, 122.5, 119.8, 119.7, 109.6, 105.7, 55.4, 31.9 ppm. IR (thin film): 1622, 1514, 1433, 1401, 1206, 1148, 1118, 1006, 804 cm^−1^; HRMS calculated for C_19_H_17_N_2_O, 289.1341, found 289.1349, [M+H]^+^.

### 1-Methyl-2-(naphthalen-1-yl)-1H-benzoimidazole (3v)

White solid (96.7 mg, 75% yield); ^1^H NMR (400 MHz, CDCl_3_): δ = 8.05 (d, *J* = 8.2 Hz.1H), 7.98 (d, *J* = 7.6 Hz.1H), 7.94–7.92 (m, 1H), 7.75 (d, *J* = 8.3 Hz.1H), 7.71 (dd, *J* = 7.0 Hz, 1.2 Hz, 1H), 7.65–7.61 (m, 1H), 7.59–7.48 (m, 3H), 7.42–7.39 (m, 2H), 3.65 (s, 3H) ppm; ^13^C NMR (100 MHz, CDCl_3_): δ = 152.9, 143.2, 135.9, 133.6, 132.2, 130.3, 128.9, 128.5, 127.8, 127.2, 126.4, 125.5, 125.1, 122.9, 122.4, 120.1, 109.6, 31.1 ppm. The ^1^H and ^13^C NMR data for this compound match the literature data^44^.

## Additional Information

**How to cite this article**: Jia, N.-N. *et al*. Copper-catalyzed Direct 2-Arylation of Benzoxazoles and Benzoimidazoles with Aryl Bromides and Cytotoxicity of Products. *Sci. Rep.* **7**, 43758; doi: 10.1038/srep43758 (2017).

**Publisher's note:** Springer Nature remains neutral with regard to jurisdictional claims in published maps and institutional affiliations.

## Supplementary Material

Supporting Information

## Figures and Tables

**Figure 1:**
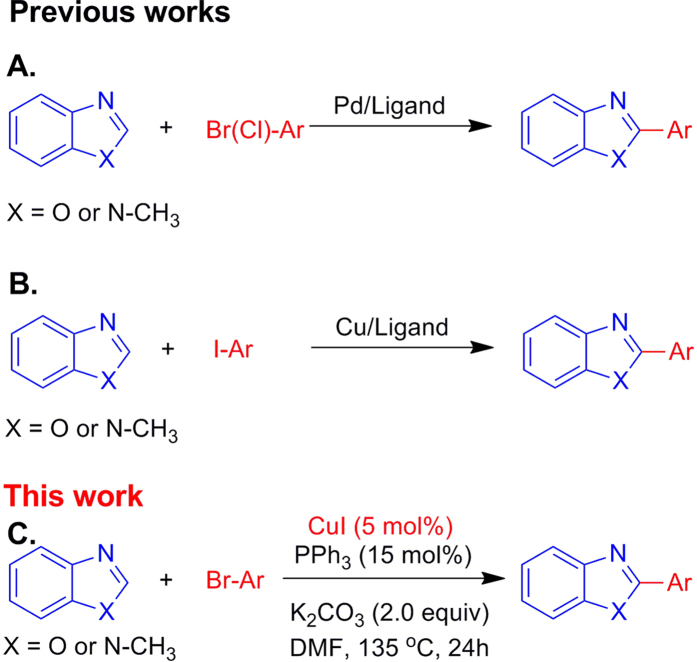
Synthesis of 2-arylbenzoxazoles.

**Table 1:**
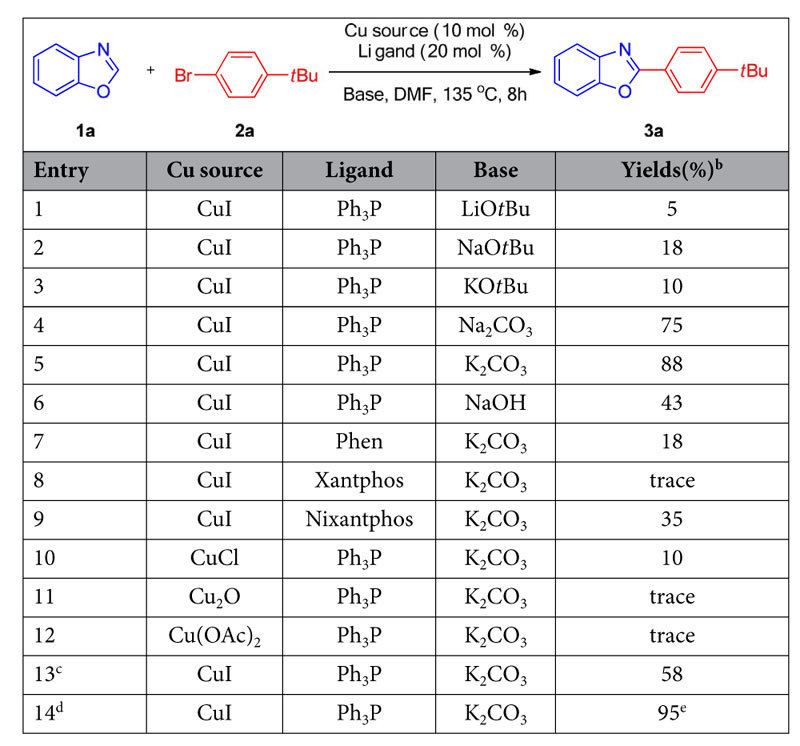
Optimization of direct 2-arylation of benzoxazole (1a) with 1-bromo-4-tert-butylbenzene (2a)^*a*^.

^*a*^Reaction conditions: **1a** (0.2 mmol), **1b** (0.24 mmol), base (0.6 mmol), Copper/Ligand (10 mol%/20 mol%), DMF (2.0 mL), 135 °C, 8 h. ^*b*^Yield determined by ^1^H-NMR spectroscopy of the crude reaction mixture.^*c*^This reaction was performed using **1a** (0.2 mmol), **1b** (0.24 mmol), K_2_CO_3_ (0.6 mmol), CuI/Ph_3_P (5 mol%/15 mol%), DMF (2.0 mL), 135 °C, 8 h.^*d*^This reaction was performed using **1a** (0.2 mmol), **1b** (0.24 mmol), K_2_CO_3_ (0.6 mmol), CuI/Ph_3_P (5 mol%/15 mol%), DMF (2.0 mL), 135^o^C, 24 h. ^*e*^Isolated yield.

**Table 2:**
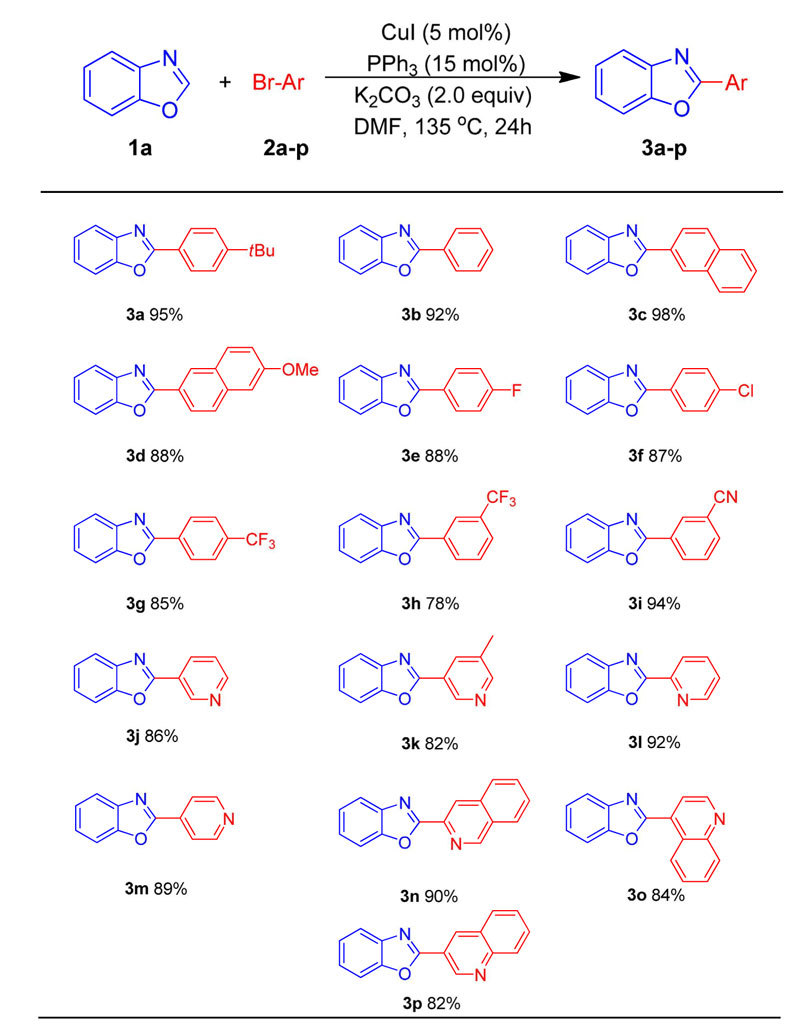
Scope of aryl bromides in direct 2-arylation of benzoxazole 1a.

**Table 3:**
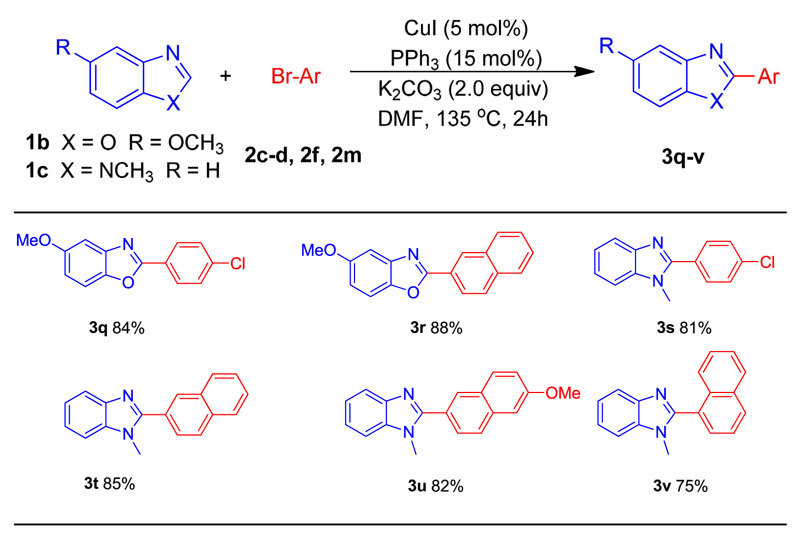
Scope of azoles in direct 2-arylation with aryl bromides.

**Table 4:** Cytotoxicity of 2-aryl benzoxazoles (3a-o) and benzoimidazoles (3p-r) and paclitaxel *in vitro*.

	IC_50_( × 10^−6^ mol/L)				
	HCT-116	HepG2	BGC-823	NCI-H1650	A2780
**3a**	>10	>10	>10	>10	>10
**3b**	>10	>10	>10	>10	>10
**3c**	>10	>10	>10	>10	>10
**3d**	>10	>10	>10	>10	>10
**3e**	>10	>10	>10	>10	>10
**3 f**	>10	>10	>10	>10	>10
**3 g**	>10	>10	>10	>10	>10
**3 h**	>10	>10	>10	>10	>10
**3i**	>10	>10	>10	>10	>10
**3j**	>10	>10	>10	>10	>10
**3k**	>10	>10	>10	>10	>10
**3 l**	>10	>10	>10	>10	>10
**3 m**	>10	>10	>10	>10	>10
**3n**	>10	>10	**8.6**	>10	>10
**3o**	>10	>10	**9.4**	>10	>10
**3p**	>10	>10	**9.1**	>10	>10
**3q**	>10	>10	>10	>10	>10
**3r**	>10	>10	>10	>10	>10
**3 s**	>10	>10	>10	>10	>10
**3t**	>10	>10	**6.5**	>10	>10
**3 u**	>10	>10	**8.5**	>10	>10
**3 v**	>10	>10	**9.2**	>10	>10
Taxol	3.16 × 10^−2^	1.78 × 10^−2^	1.17 × 10^−3^	6.95 × 10^−2^	3.30 × 10^−2^

HCT-116: human colon cancer cell; HepG2: human liver cancer cell; BGC-823: human stomach cancer cell; NCI-H1650: human non-small cell lung cancer cell; A2780: human ovarian cancer cell.
