# Supplementary material for: Copper-catalyzed Direct 2-Arylation of Benzoxazoles and Benzoimidazoles with Aryl Bromides and Cytotoxicity of Products
Source: Sci Rep. 2017 Mar 3;7:43758. doi: 10.1038/srep43758 (PMC5335610; doi:10.1038/srep43758)
Supplement: Supporting Information [file srep43758-s1.pdf]

SUPPORTING INFORMATION

Copper-catalyzed Direct 2-Arylation of Benzoxazoles and Benzoimidazoles with Aryl Bromides and Cytotoxicity of Products

Nan-Nan Jia,[1,2,§] Xin-Chuan Tian,[2,§] Xiao-Xia Qu,[2] Xing-Xiu Chen,[2] Ya-Nan Cao,[2] Yun-Xin Yao,[2] Feng Gao,[*,1,2] and Xian-Li Zhou*[,1]

[1] School of Life Science and Engineering, Southwest Jiaotong University, Chengdu 610031, P.R. China

[2] Department of Chinese Traditional Herbal, Agronomy College, Sichuan Agriculture University, Chengdu 611130, P. R. China

[§]N.-N. J. and X.-C. T. contributed equally.

*Corresponding Author

Email: gaof@swjtu.edu.cn (F. Gao); Zhouxl@swjtu.edu.cn (X.-L. Zhou)

CONTENTS

1 X-ray crystallographic data of Cu(PPh3)3I (**4**)……………………………….2

2 ^{1}H NMR and ^{13}C NMR spectra of **3**………………………………………..3

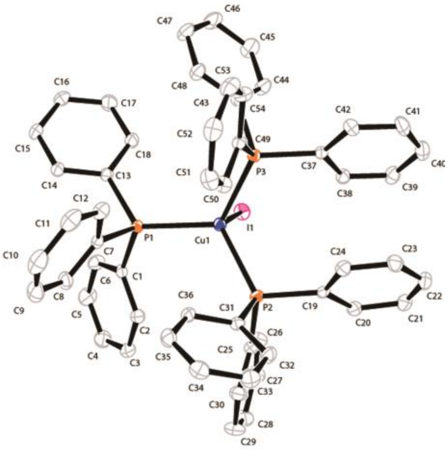

Identification code	150616_s1_qxx_m
Empirical formula	$C_{60}H_{59}CuIN_2O_2P_3$
Formula weight	1123.44
Temperature/K	293.15
Crystal system	triclinic
Space group	P-1
a/Å	13.2763(6)
b/Å	14.4028(5)
c/Å	14.8610(4)
α/°	71.280(3)
β/°	85.073(3)
γ/°	88.802(3)
Volume/Å^3	2681.31(16)
Z	2
ρ_{calc}mg/mm^3	1.391
m/mm^{-1}	1.117
F(000)	1152.0
Crystal size/mm^3	0.32 × 0.28 × 0.26
2Θ range for data collection	5.84 to 52.74°
Index ranges	-16 ≤ h ≤ 15, -17 ≤ k ≤ 17, -18 ≤ l ≤ 15
Reflections collected	22465
Independent reflections	10946[R(int) = 0.0252]
Data/restraints/parameters	10946/0/626
Goodness-of-fit on F^2	1.034
Final R indexes [I>=2σ (I)]	R_1 = 0.0400, wR_2 = 0.0883
Final R indexes [all data]	R_1 = 0.0614, wR_2 = 0.0983
Largest diff. peak/hole / e Å^{-3}	0.56/-0.44

2-(4-*tert*-Butylphenyl)benzoxazole(**3a**)

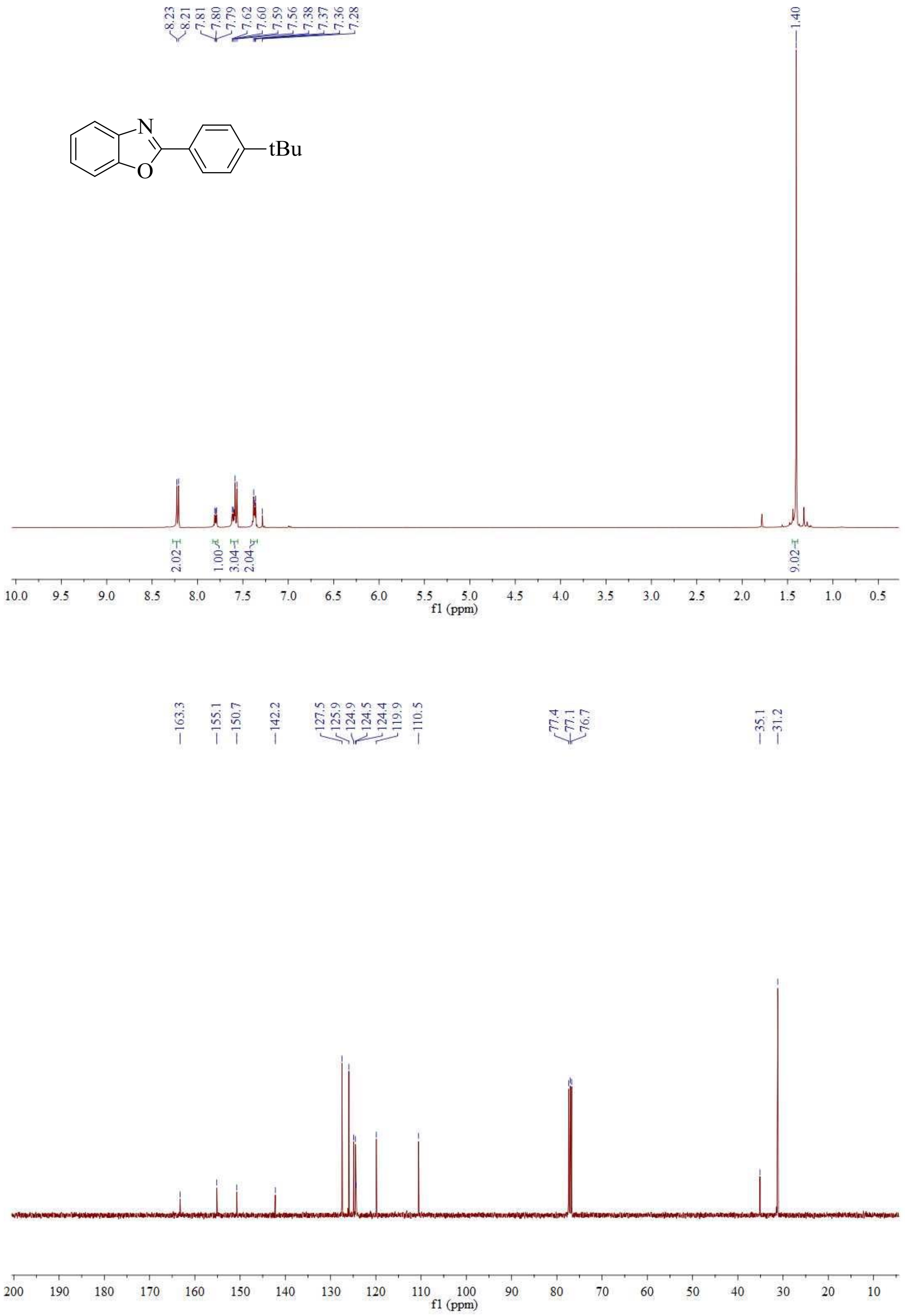

Fig. S1 ^{1}H (400 MHz) and ^{13}C {^{1}H} (100 MHz) NMR spectra of **3a** in $CDCl_3$

2-Phenylbenzoxazole (3b)

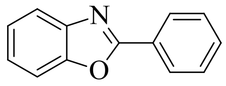

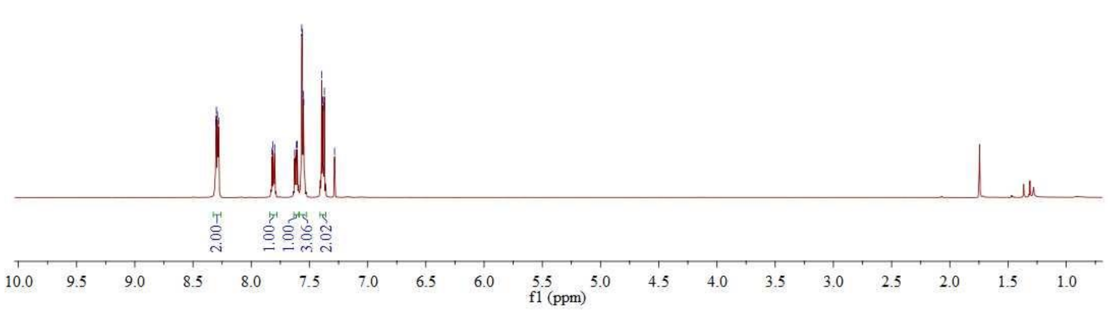

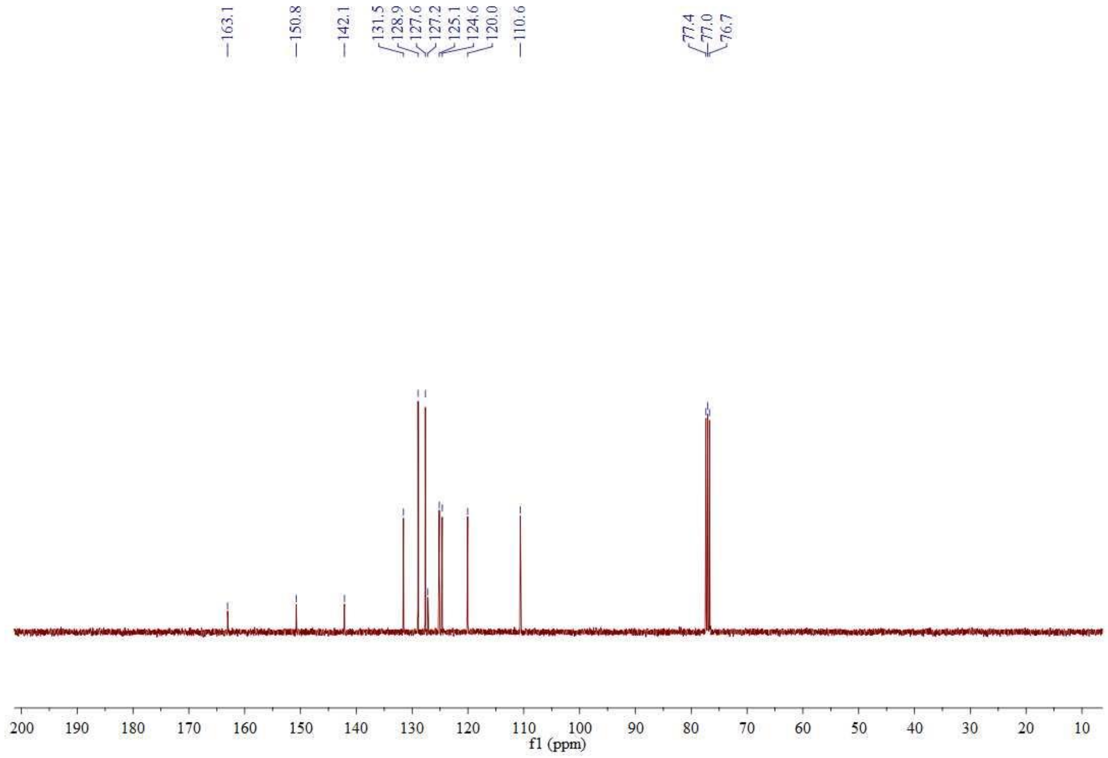

Fig. S2 ^{1}H (400 MHz) and ^{13}C {^{1}H} (100 MHz) NMR spectra of **3b** in $CDCl_3$

2-(naphthalen-2-yl)benzoxazole (3c)

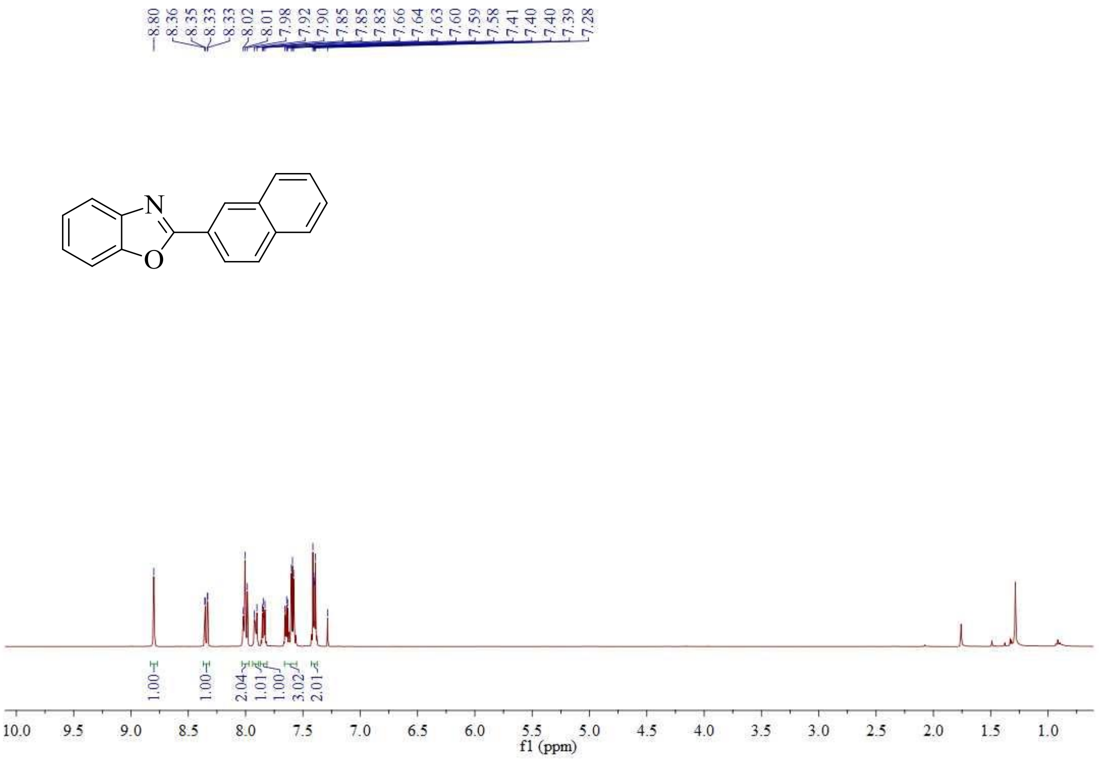

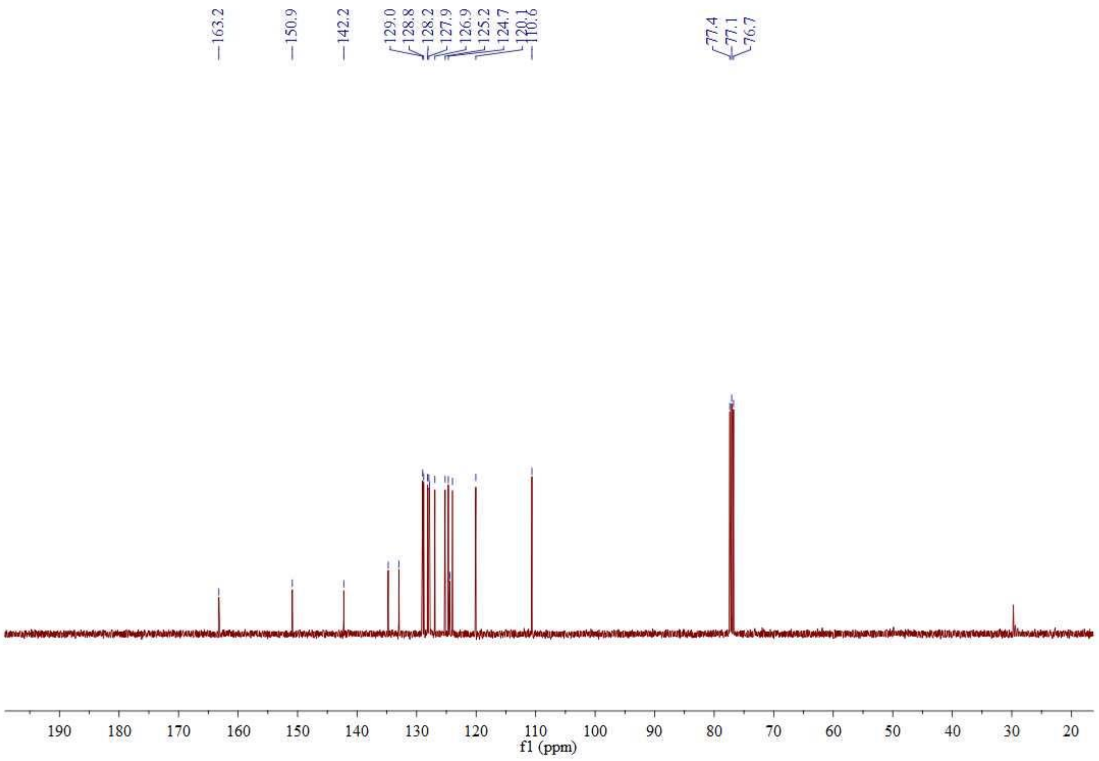

Fig. S3 ^{1}H (400 MHz) and ^{13}C {^{1}H} (100 MHz) NMR spectra of **3c** in $CDCl_3$

2-(6-methoxynaphthalen-2-yl)benzoxazole (3d)

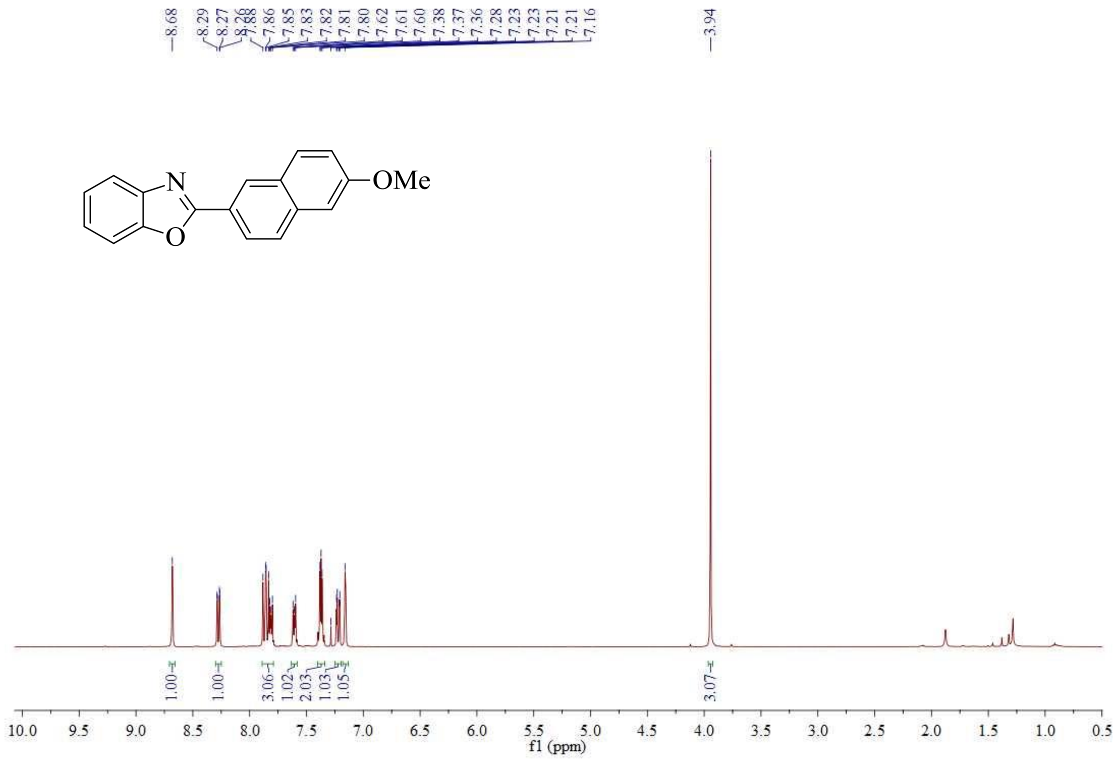

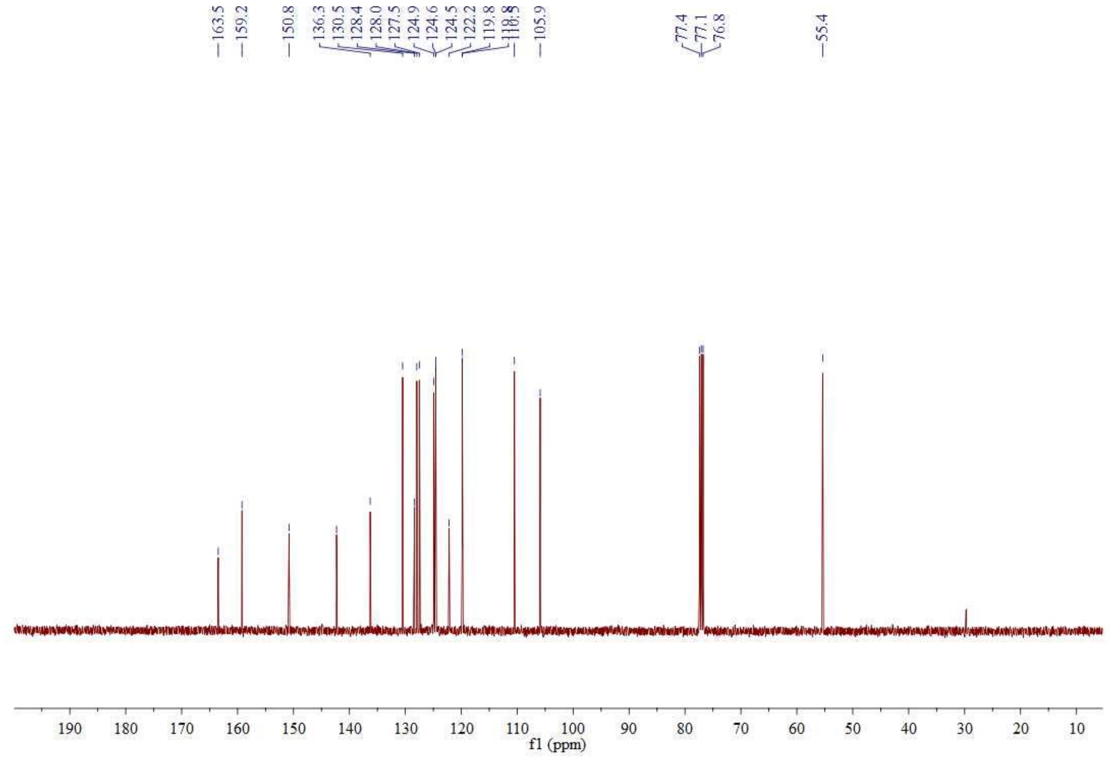

Fig. S4 ^{1}H (400 MHz) and ^{13}C {^{1}H} (100 MHz) NMR spectra of **3d** in $CDCl_3$

2-(4-fluorophenyl)benzoxazole (3e)

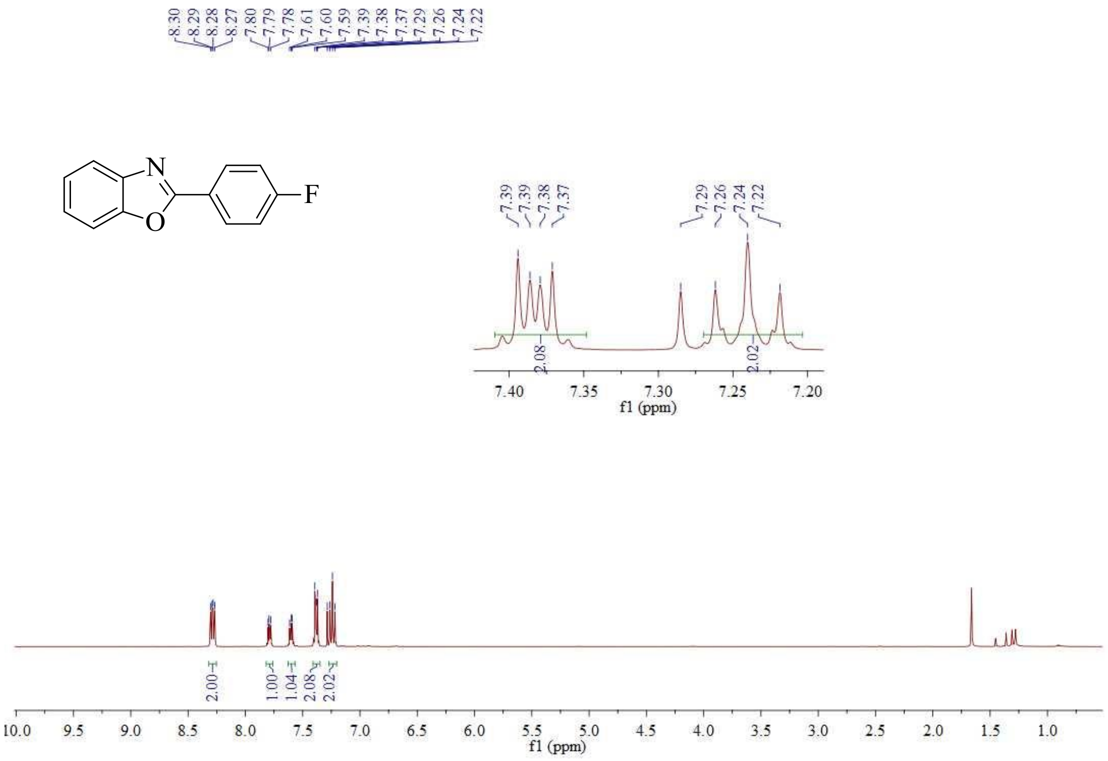

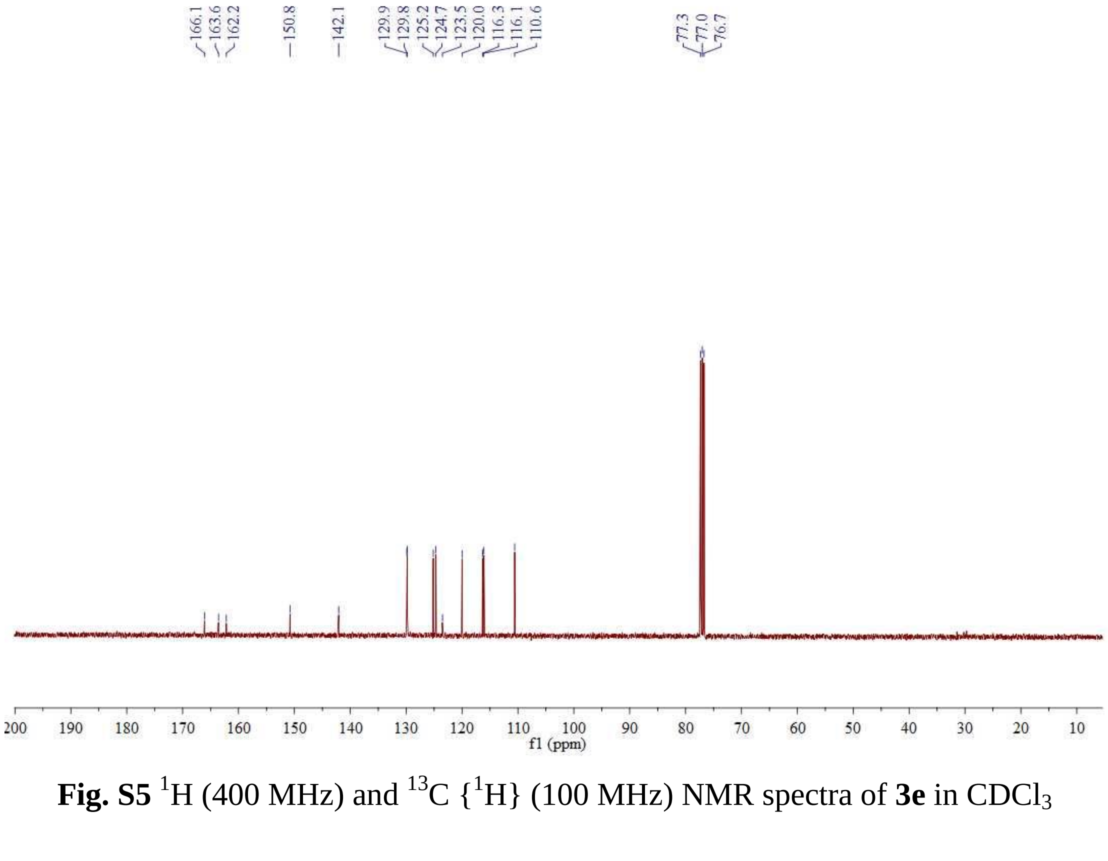

Fig. S5 ^{1}H (400 MHz) and ^{13}C {^{1}H} (100 MHz) NMR spectra of **3e** in $CDCl_3$

2-(4-chlorophenyl)benzoxazole (**3f**)

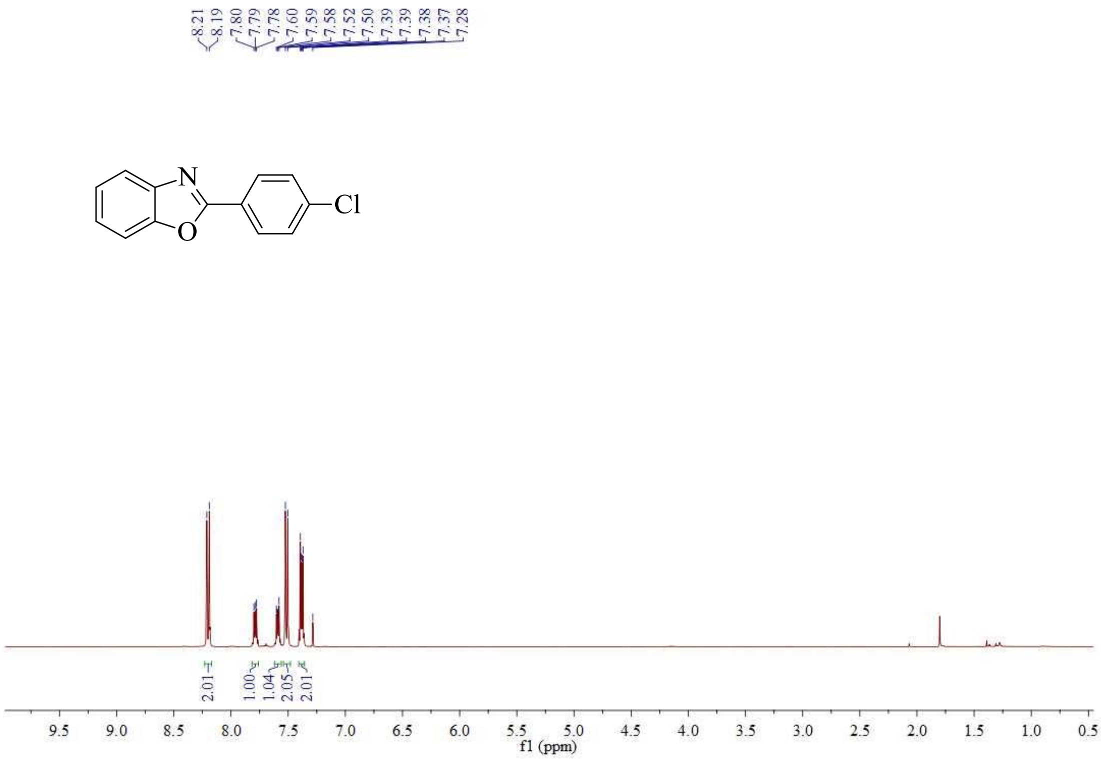

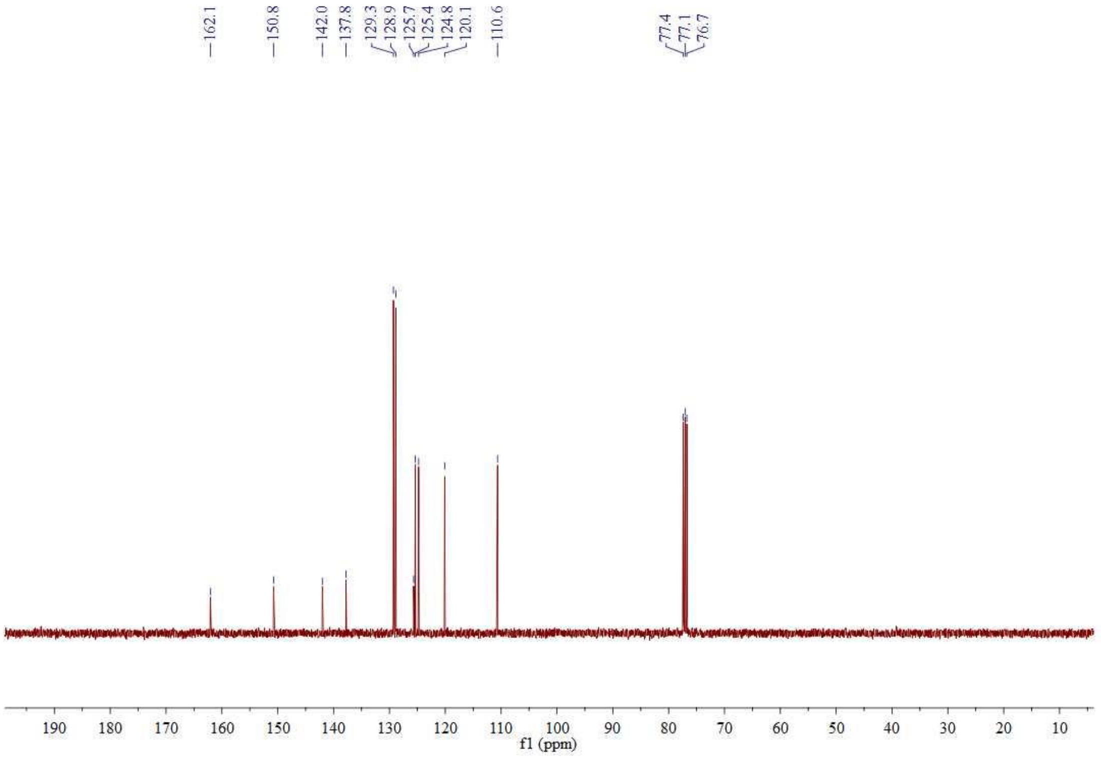

Fig. S6 ^{1}H (400 MHz) and ^{13}C {^{1}H} (100 MHz) NMR spectra of **3f** in $CDCl_3$

2-(4-(trifluoromethyl)phenyl)benzoxazole(3g)

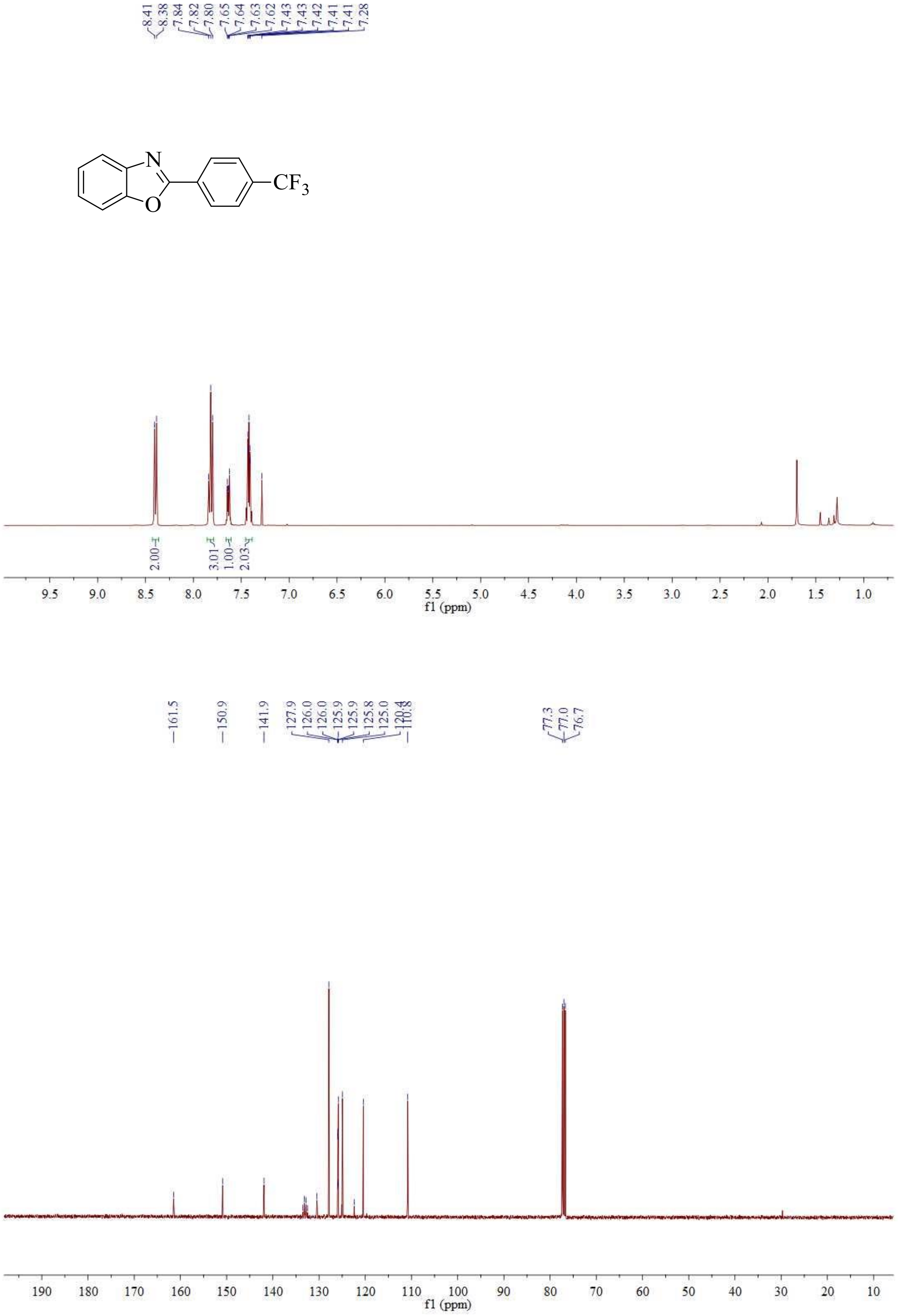

Fig. S7 ^{1}H (400 MHz) and ^{13}C {^{1}H} (100 MHz) NMR spectra of **3g** in $CDCl_3$

2-(3-(trifluoromethyl)phenyl)benzoxazole (**3h**)

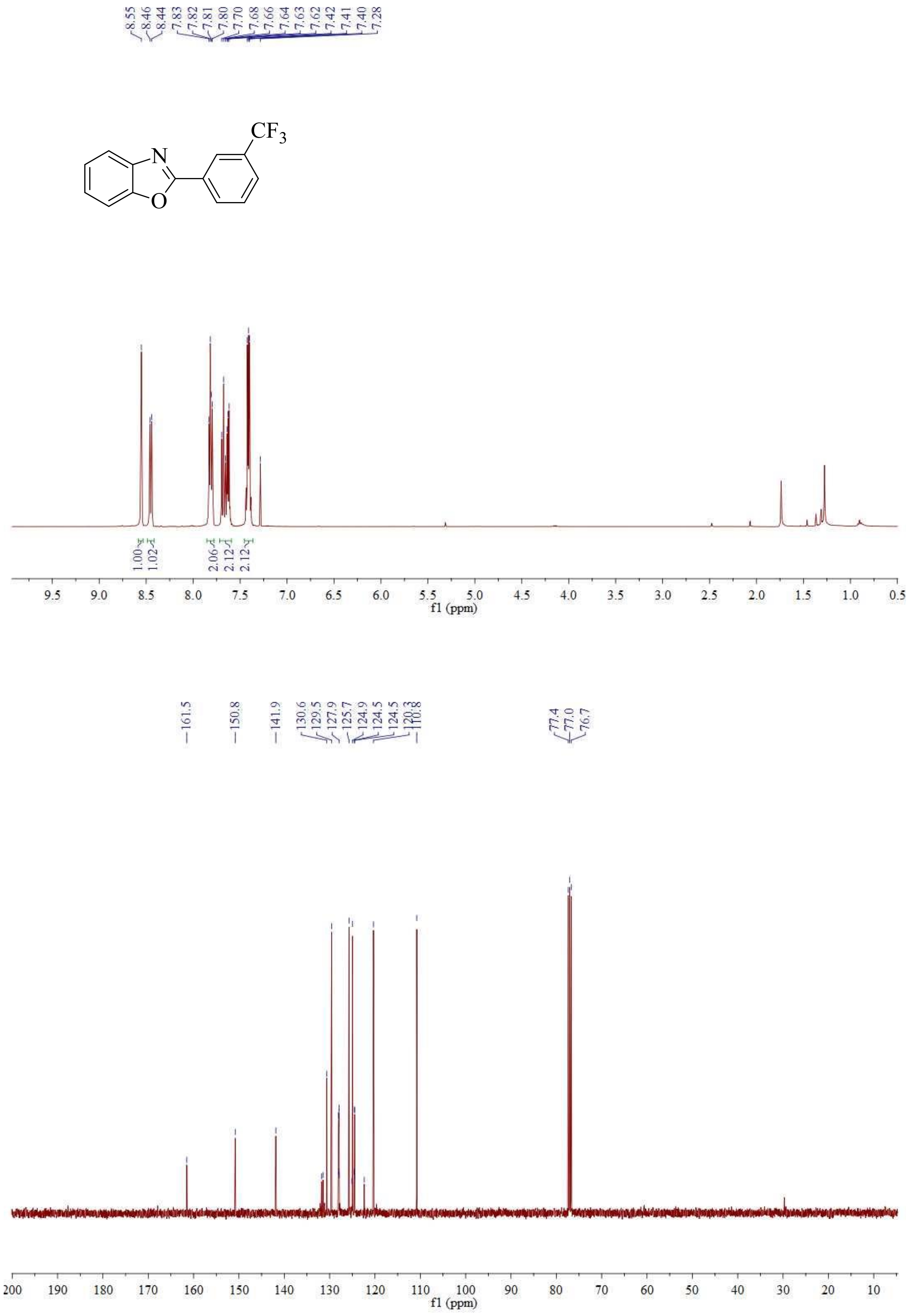

Fig. S8 ^{1}H (400 MHz) and ^{13}C {^{1}H} (100 MHz) NMR spectra of **3h** in $CDCl_3$

3-(benzoxazol-2-yl)benzonitrile (**3i**)

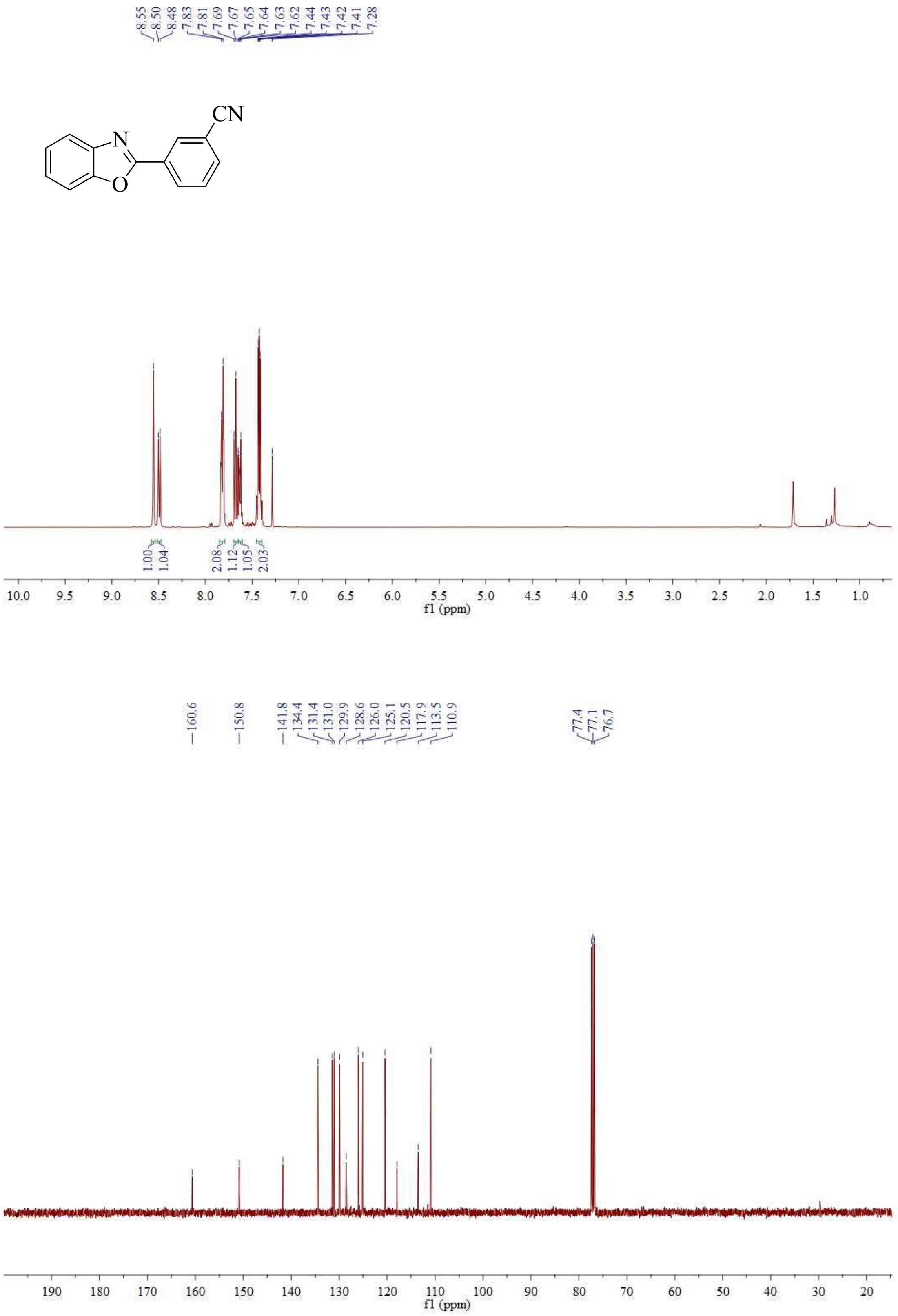

Fig. S9 ^{1}H (400 MHz) and ^{13}C {^{1}H} (100 MHz) NMR spectra of **3i** in $CDCl_3$

2-(pyridin-3-yl)benzoxazole(3j)

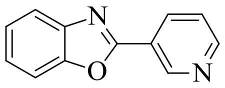

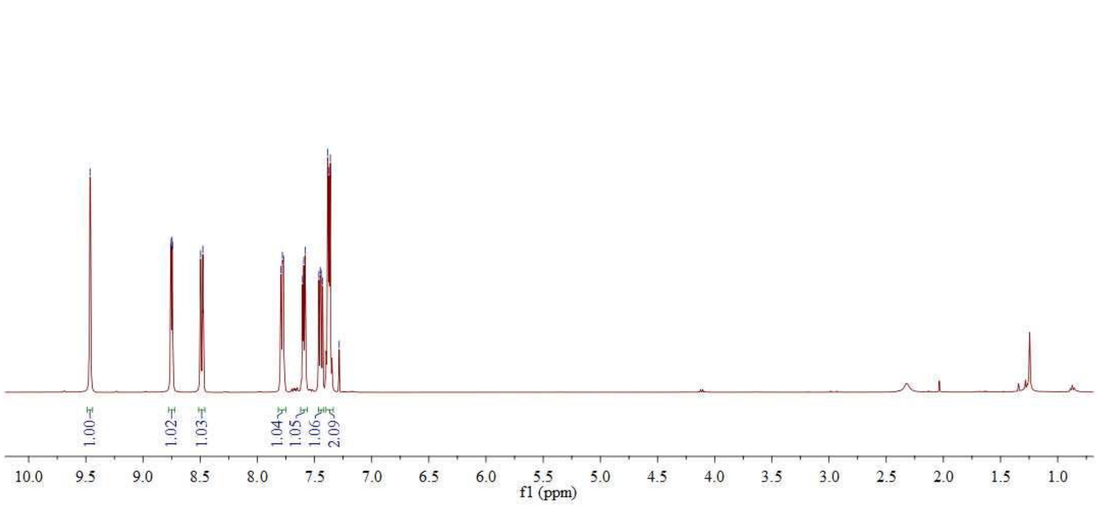

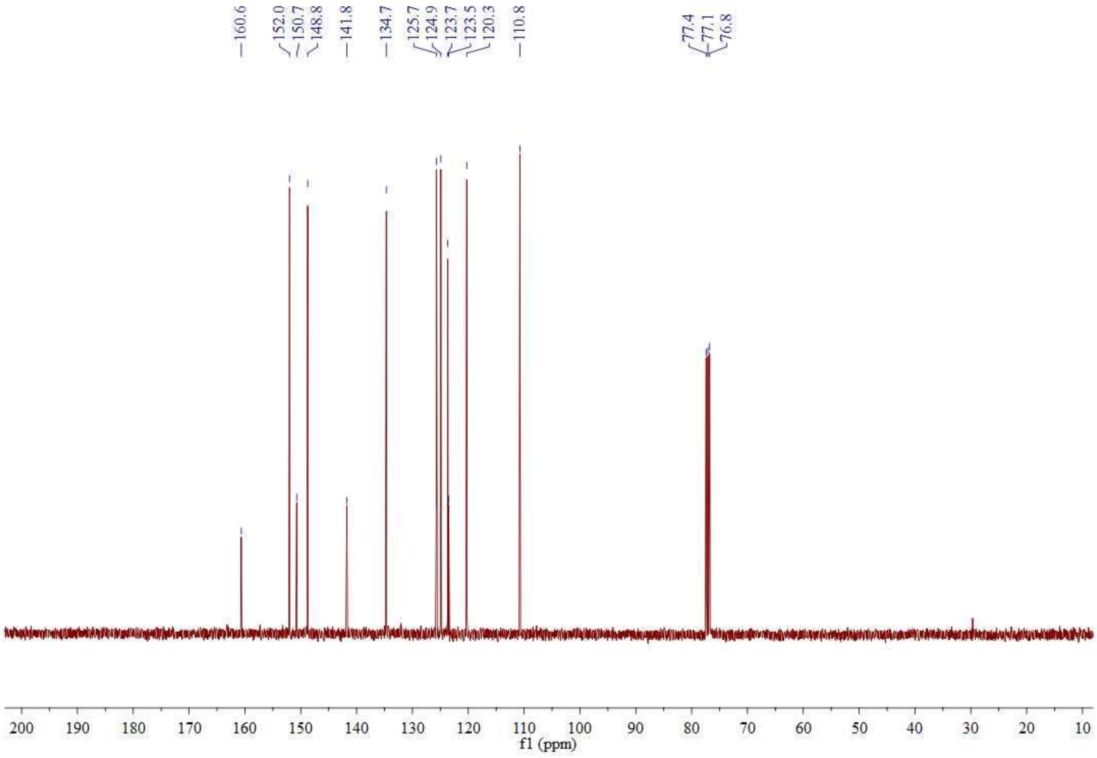

Fig. S10 ^{1}H (400 MHz) and ^{13}C {^{1}H} (100 MHz) NMR spectra of **3j** in $CDCl_3$

2-(5-methylpyridin-3-yl)benzoxazole (3k)

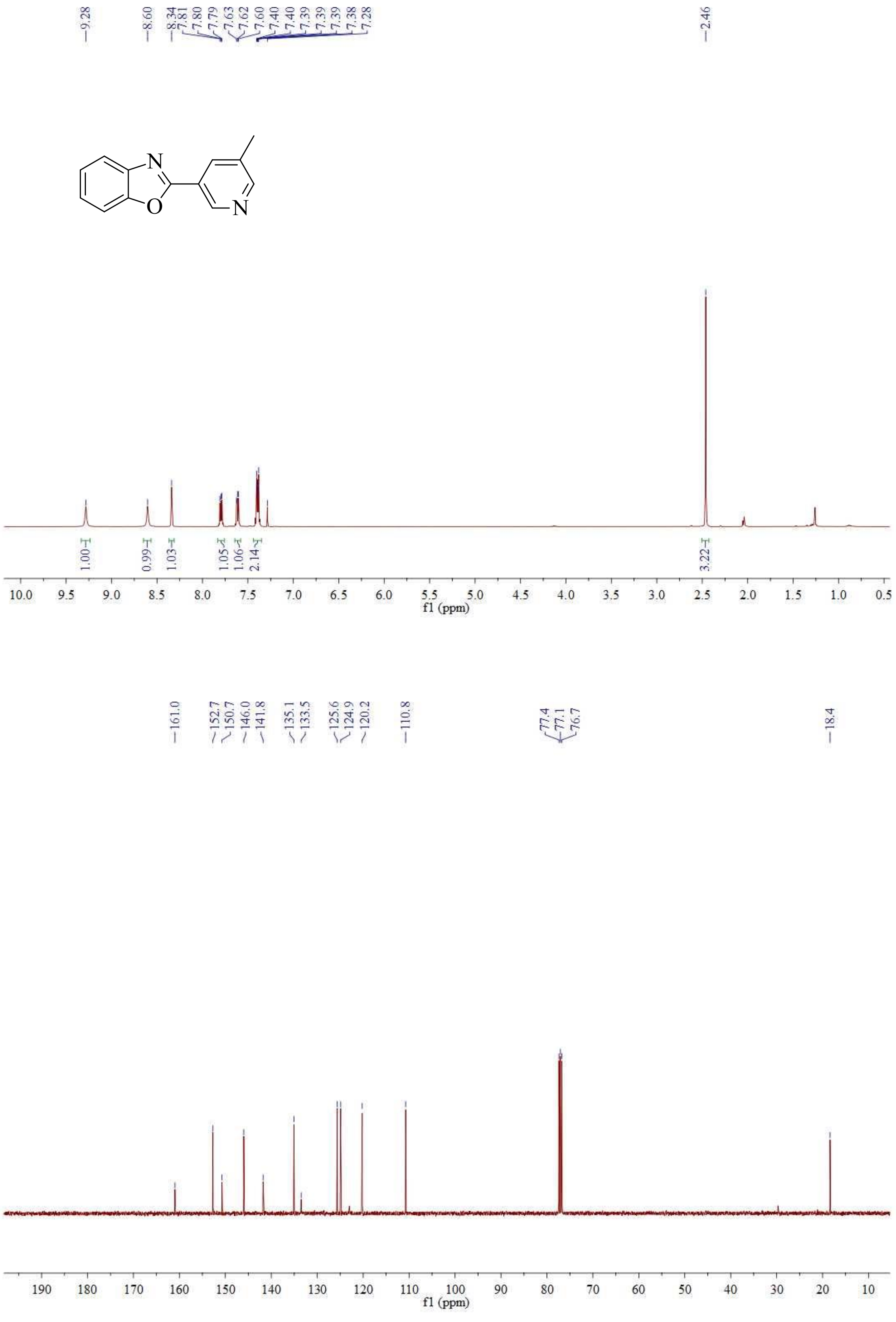

Fig. S11 ^{1}H (400 MHz) and ^{13}C {^{1}H} (100 MHz) NMR spectra of **3k** in $CDCl_3$

2-(pyridin-2-yl)benzoxazole (3l)

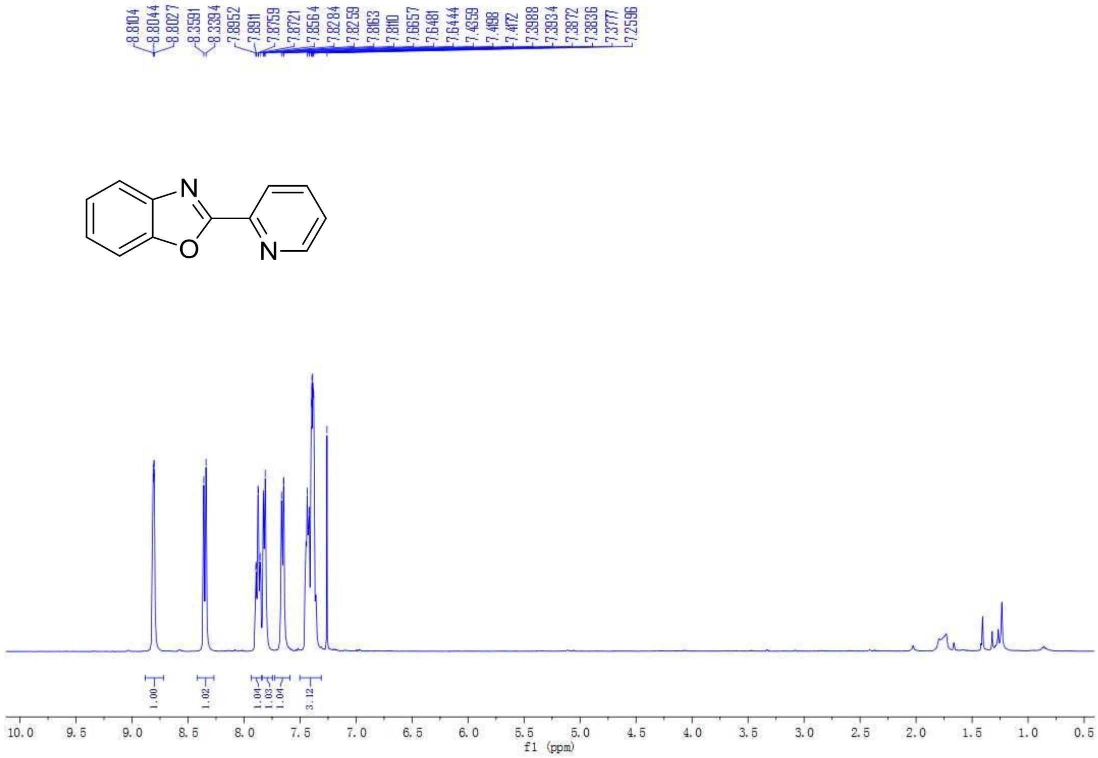

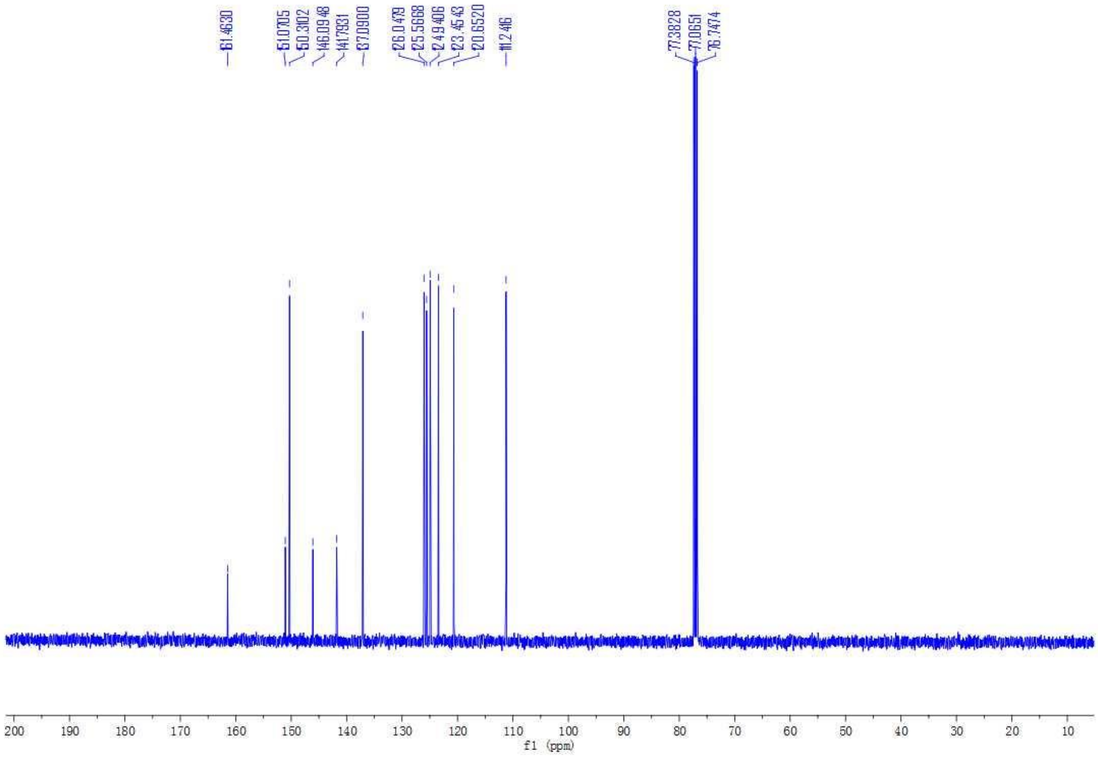

Fig. S12 ^{1}H (400 MHz) and ^{13}C {^{1}H} (100 MHz) NMR spectra of **3l** in $CDCl_3$

2-(pyridin-4-yl)benzo[d]oxazole (3m)

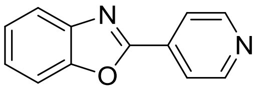

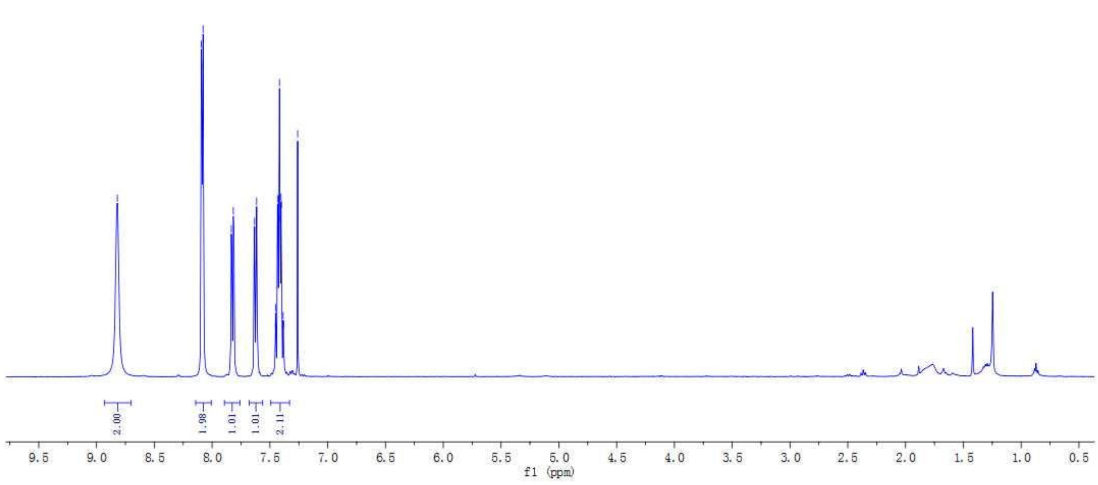

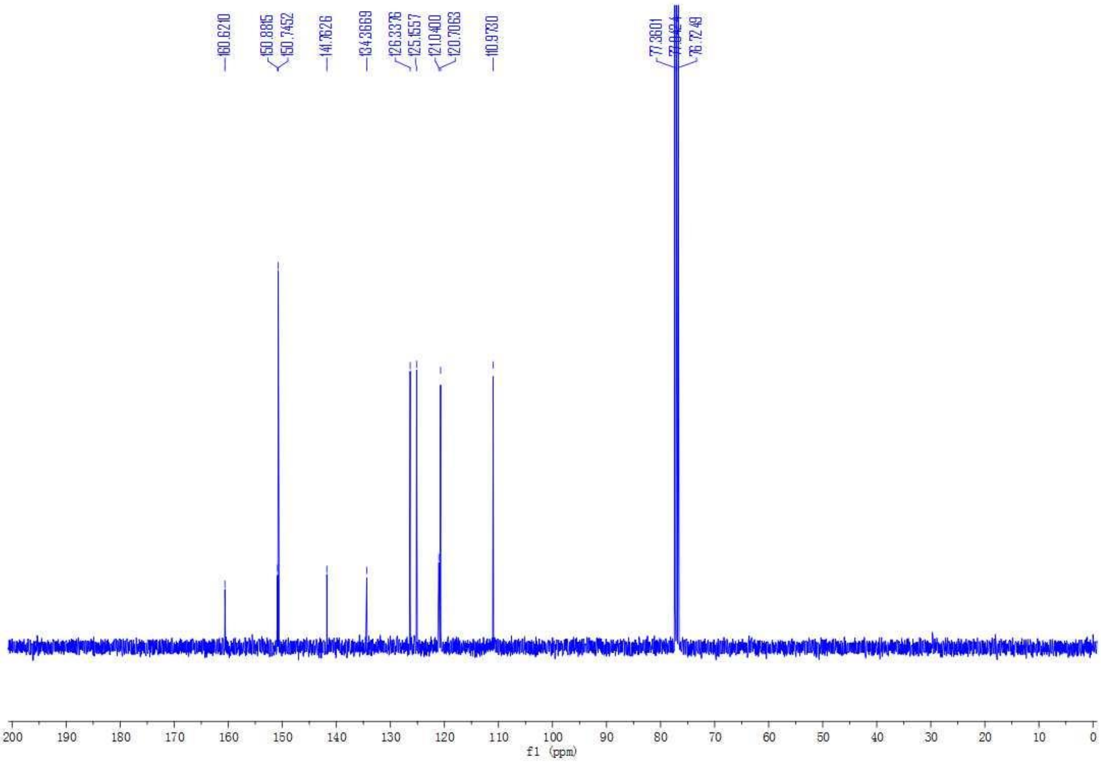

Fig. S13 ^{1}H (400 MHz) and ^{13}C {^{1}H} (100 MHz) NMR spectra of **3m** in $CDCl_3$

2-(isoquinolin-3-yl)benzoxazole (3n)

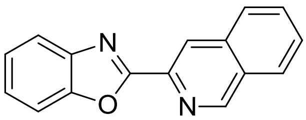

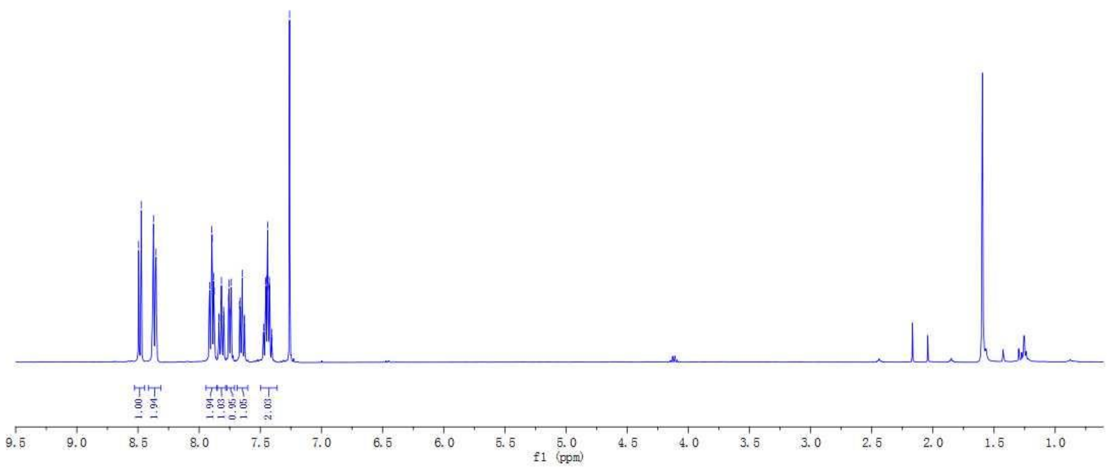

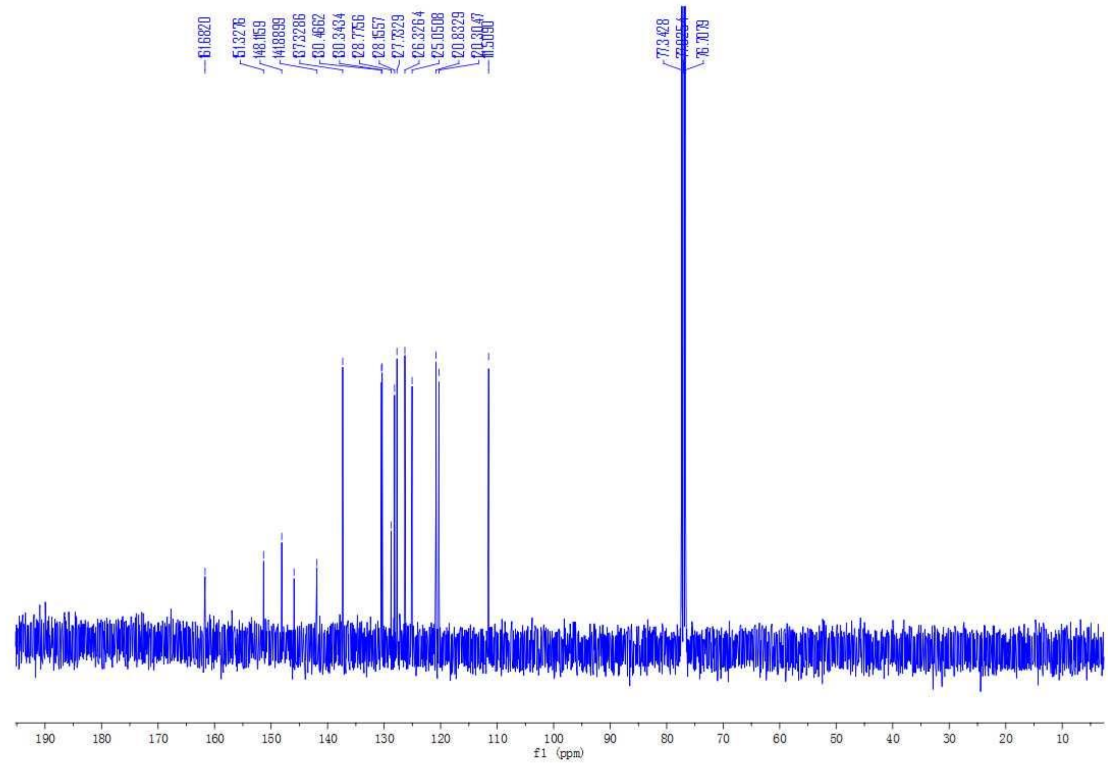

Fig. S14 ^{1}H (400 MHz) and ^{13}C {^{1}H} (100 MHz) NMR spectra of **3n** in $CDCl_3$

2-(Quinolin-4-yl)benzoxazole (3o)

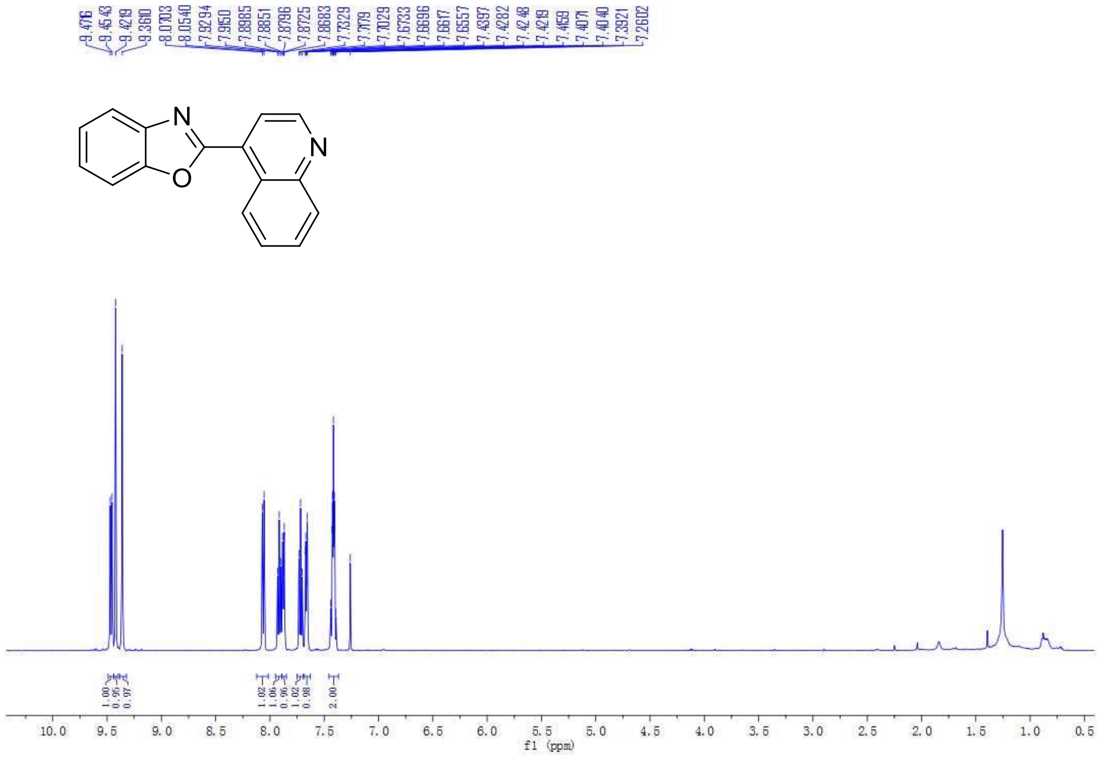

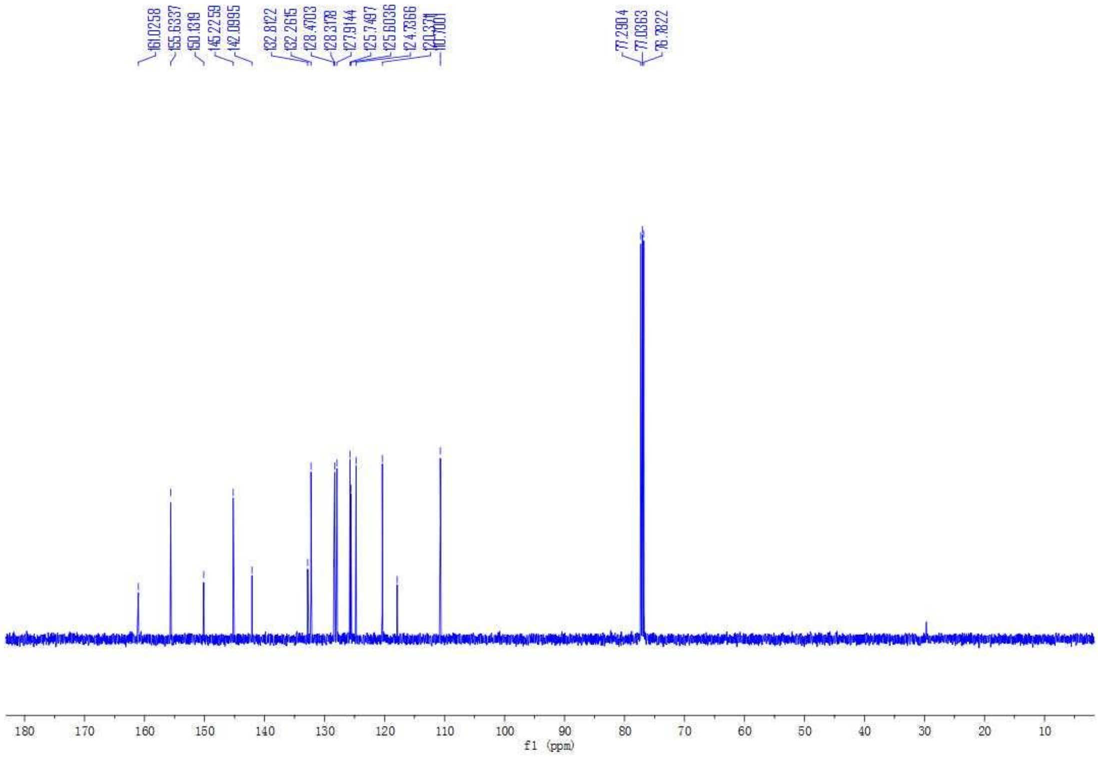

Fig. S15 ^{1}H (400 MHz) and ^{13}C {^{1}H} (100 MHz) NMR spectra of **3o** in $CDCl_3$

3-benzooxazol-2-ylquinoline (3p)

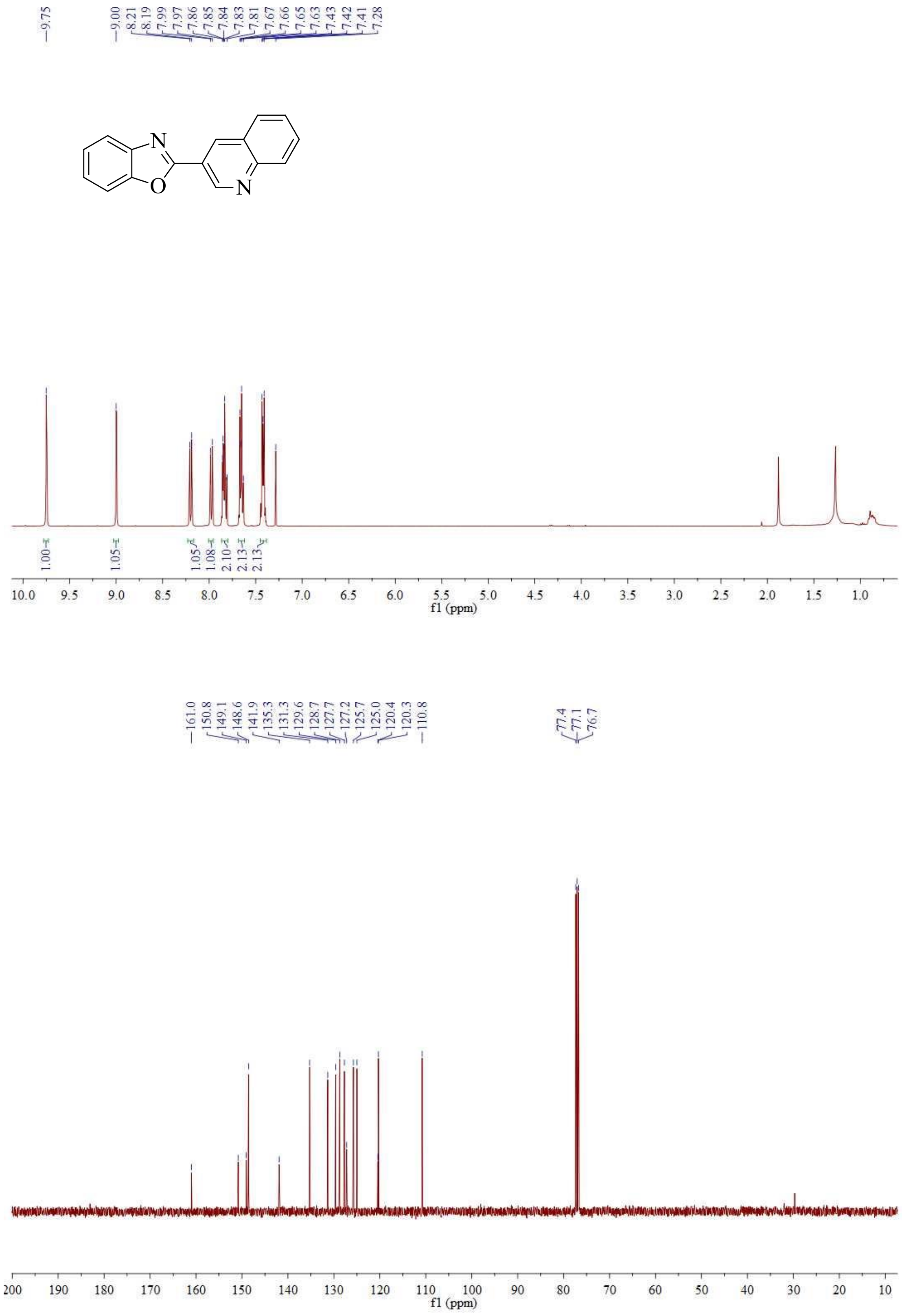

Fig. S16 ^{1}H (400 MHz) and ^{13}C {^{1}H} (100 MHz) NMR spectra of **3p** in $CDCl_3$

2-(4-chlorophenyl)-5-methoxybenzoxazole (3q)

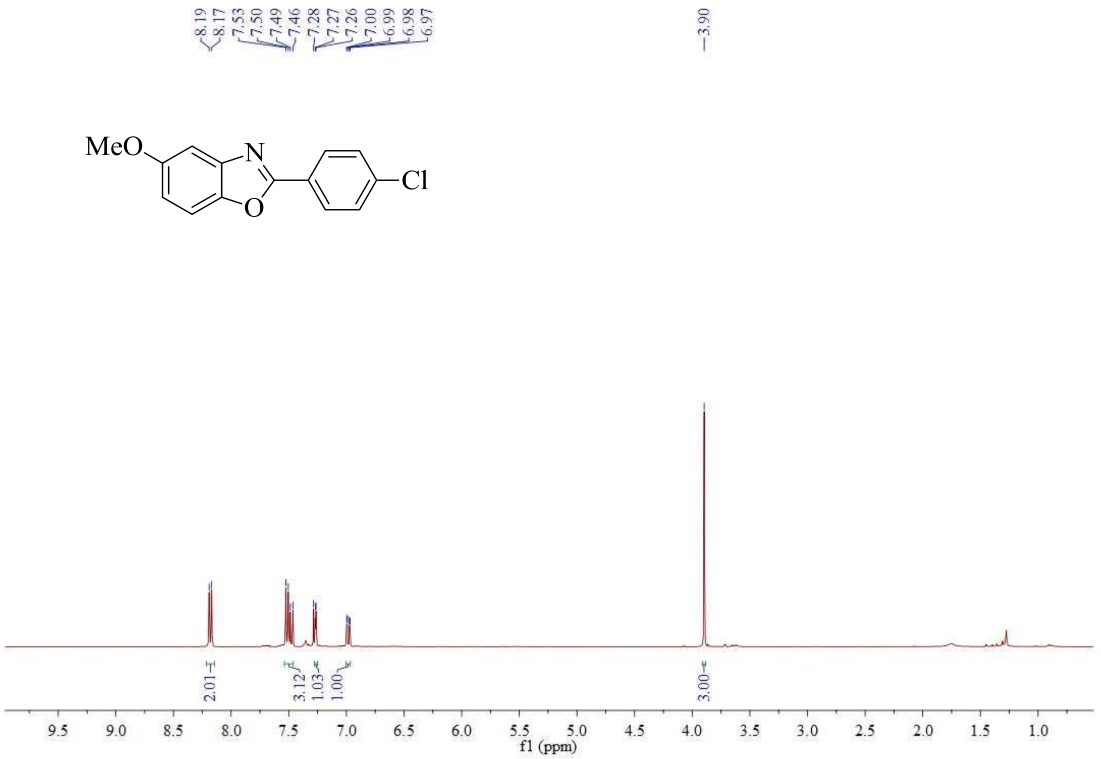

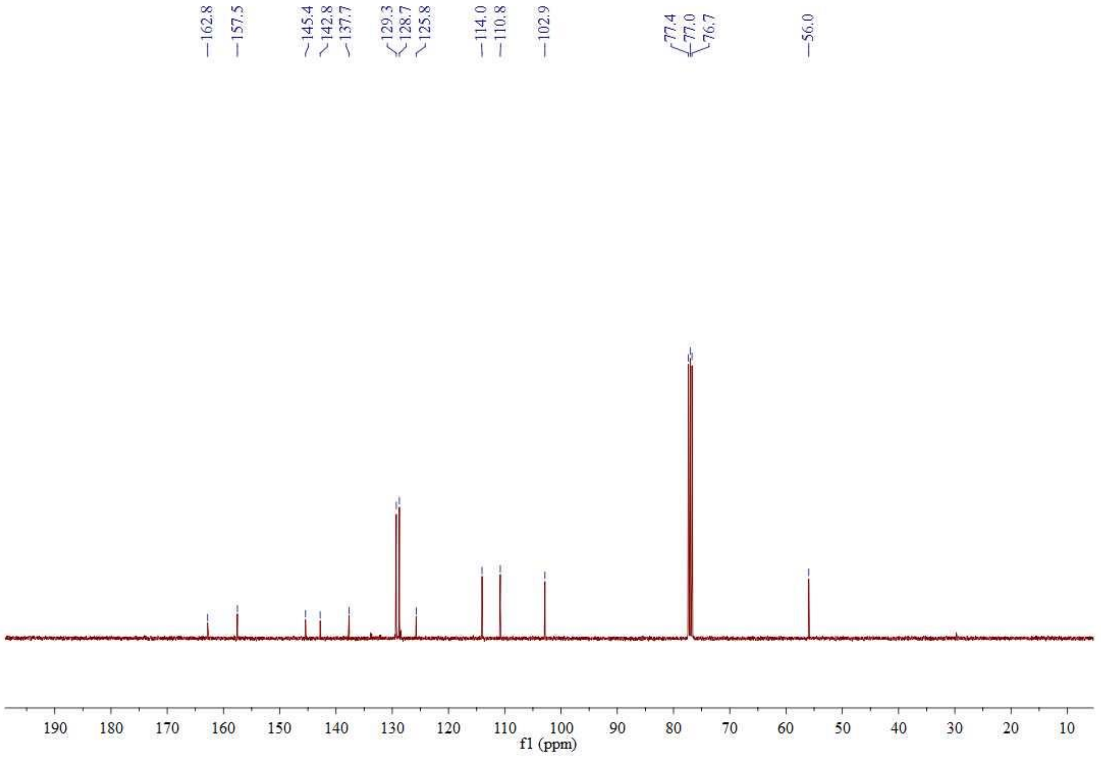

Fig. S17 ^{1}H (400 MHz) and $^{13}C\ \{^{1}H\}$ (100 MHz) NMR spectra of **3q** in $CDCl_3$

5-methoxy-2-(naphthalen-2-yl)benzoxazole (3r)

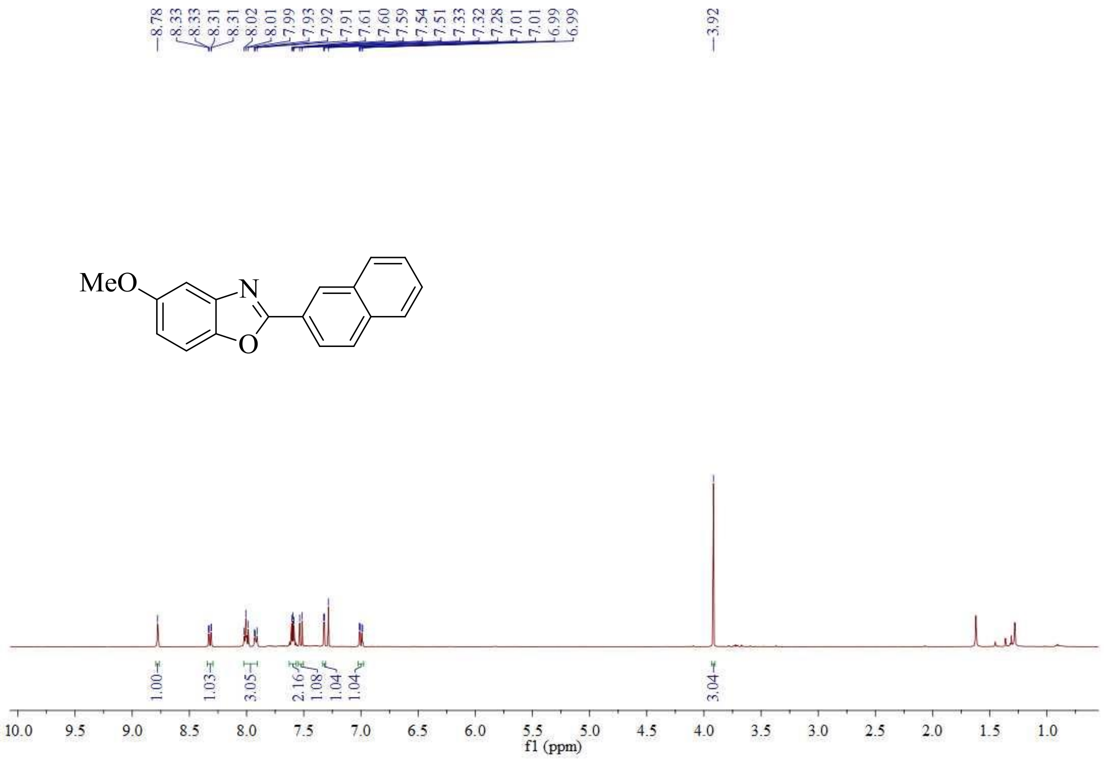

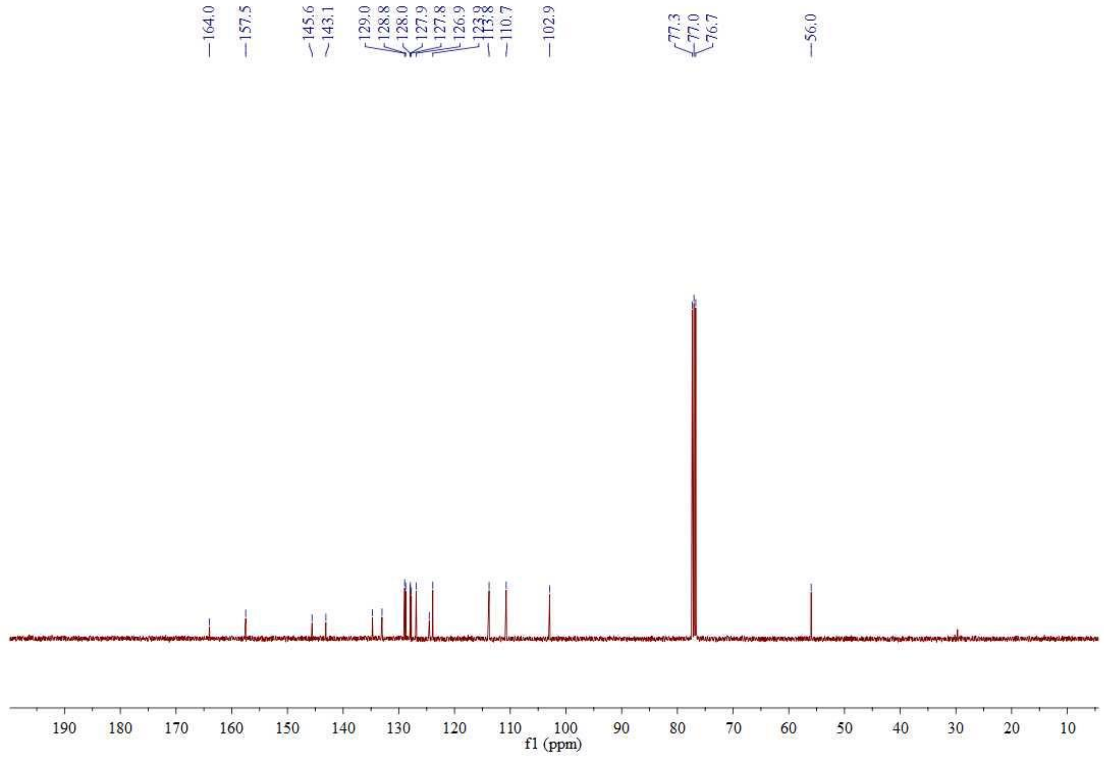

Fig. S18 ^{1}H (400 MHz) and ^{13}C {^{1}H} (100 MHz) NMR spectra of **3r** in $CDCl_3$

2-(4-Chlorophenyl)-1-methyl-1H-benzoimidazole(3s)

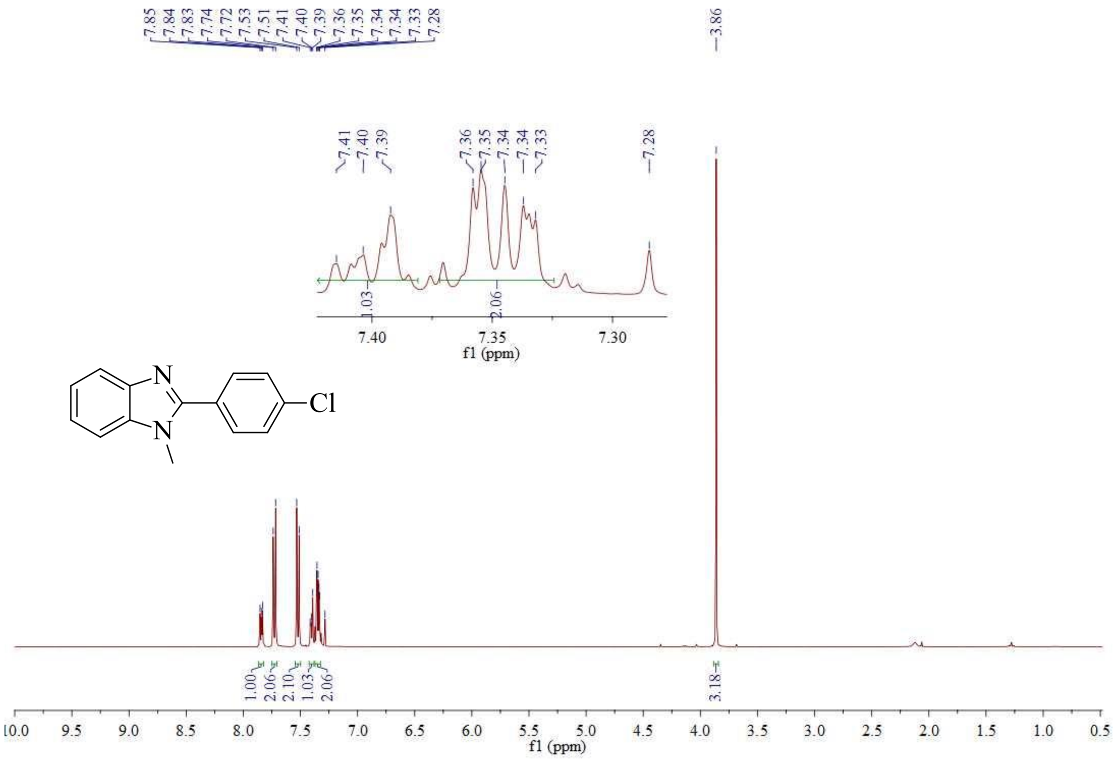

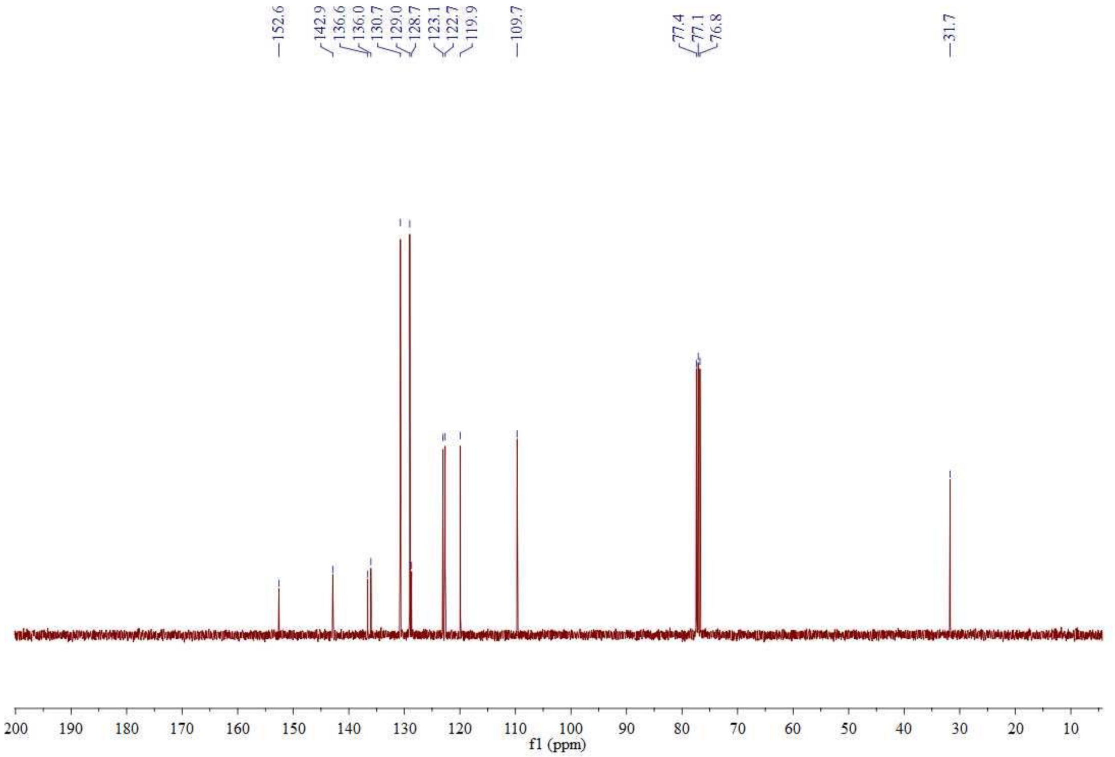

Fig. S19 ^{1}H (400 MHz) and ^{13}C {^{1}H} (100 MHz) NMR spectra of **3s** in $CDCl_3$

1-methyl-2-(naphthalen-2-yl)-1H-benzoimidazole (3t)

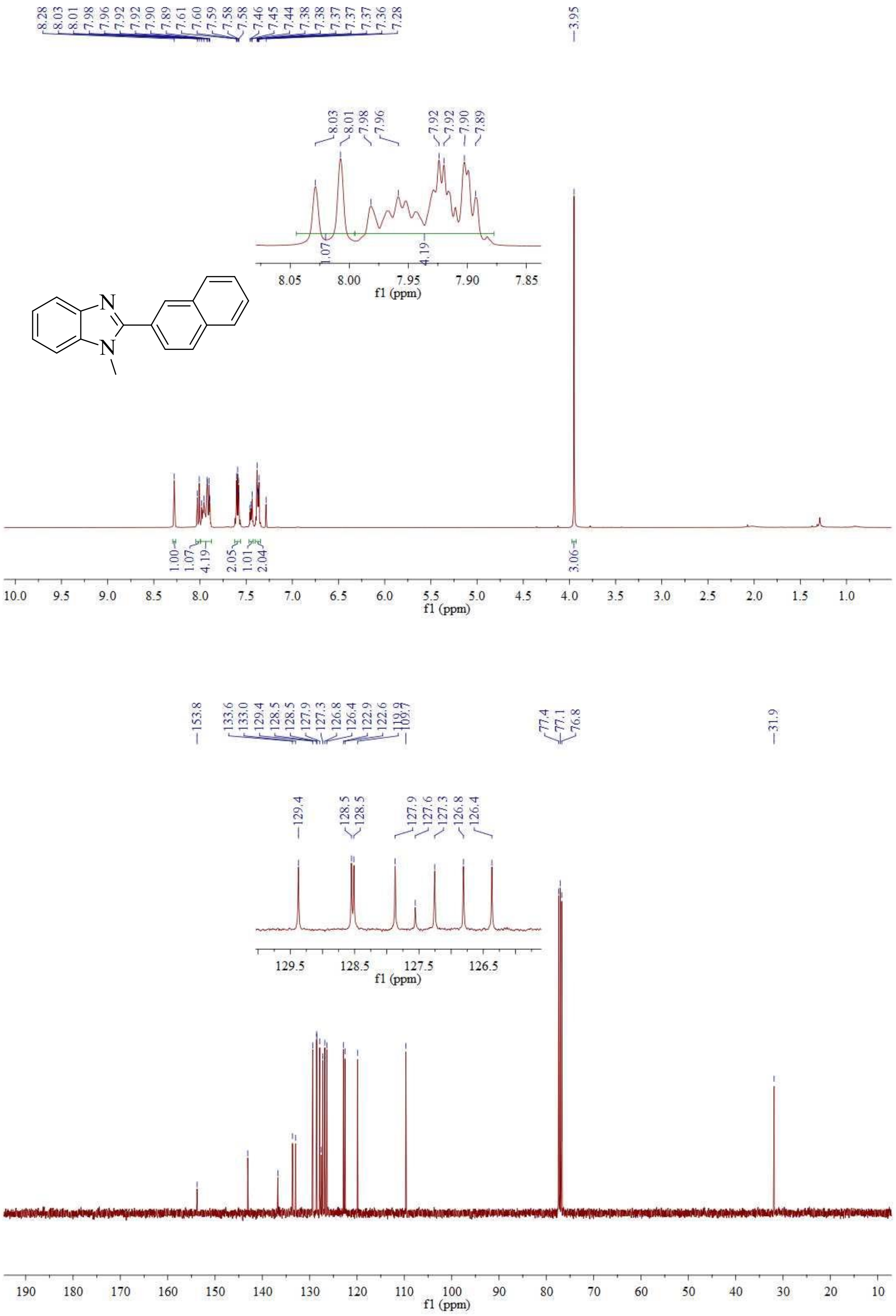

Fig. S20 ^{1}H (400 MHz) and ^{13}C {^{1}H} (100 MHz) NMR spectra of **3t** in $CDCl_3$

2-(6-methoxynaphthalen-2-yl)-1-methyl-1H-benzoimidazole (2u)

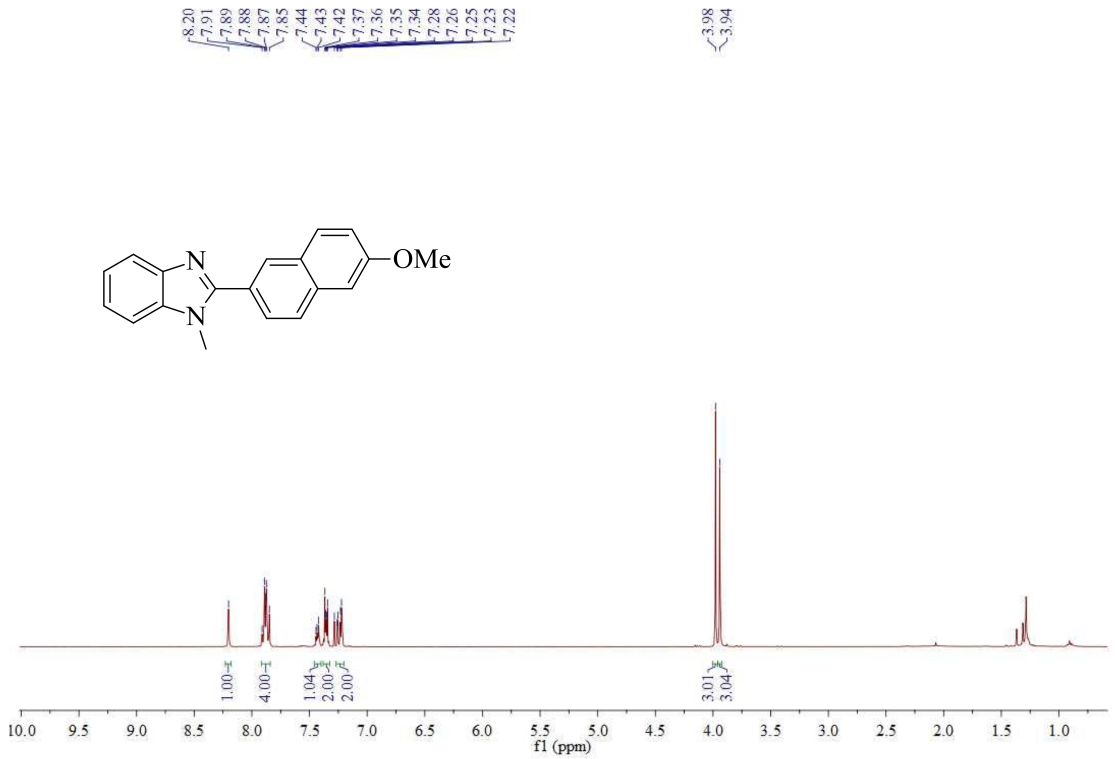

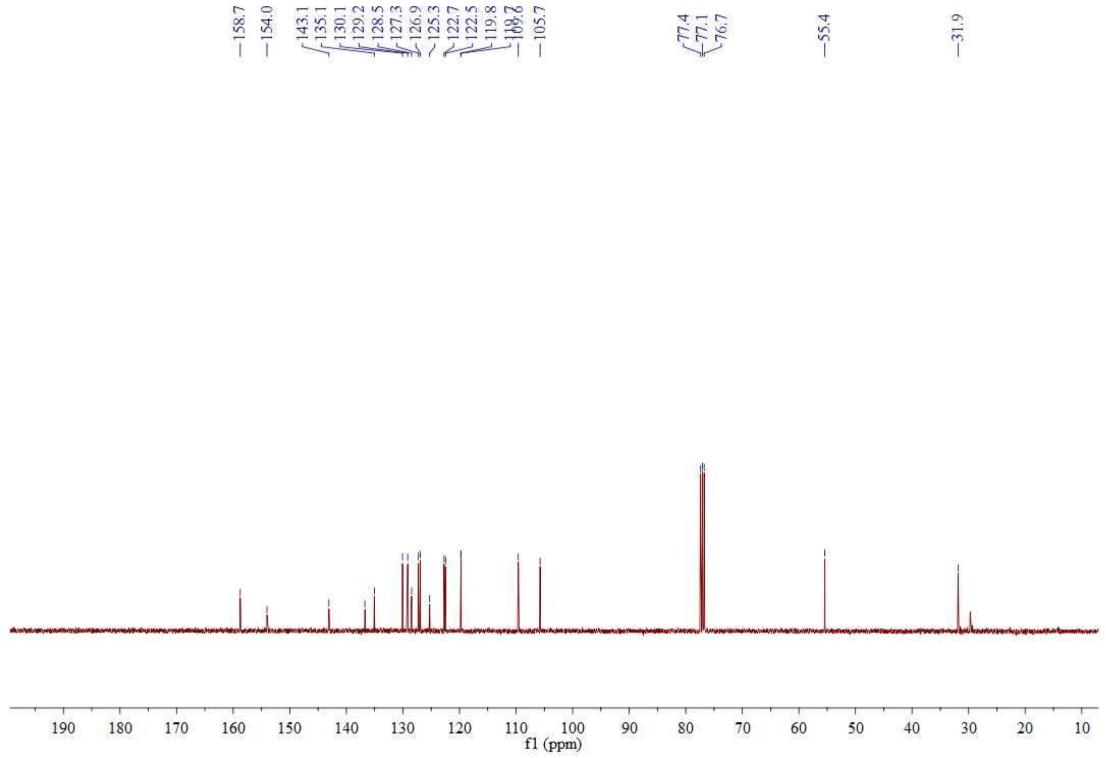

Fig. S21 ^{1}H (400 MHz) and ^{13}C {^{1}H} (100 MHz) NMR spectra of **3u** in $CDCl_3$

1-methyl-2-(naphthalen-1-yl)-1H-benzoimidazole (3v)

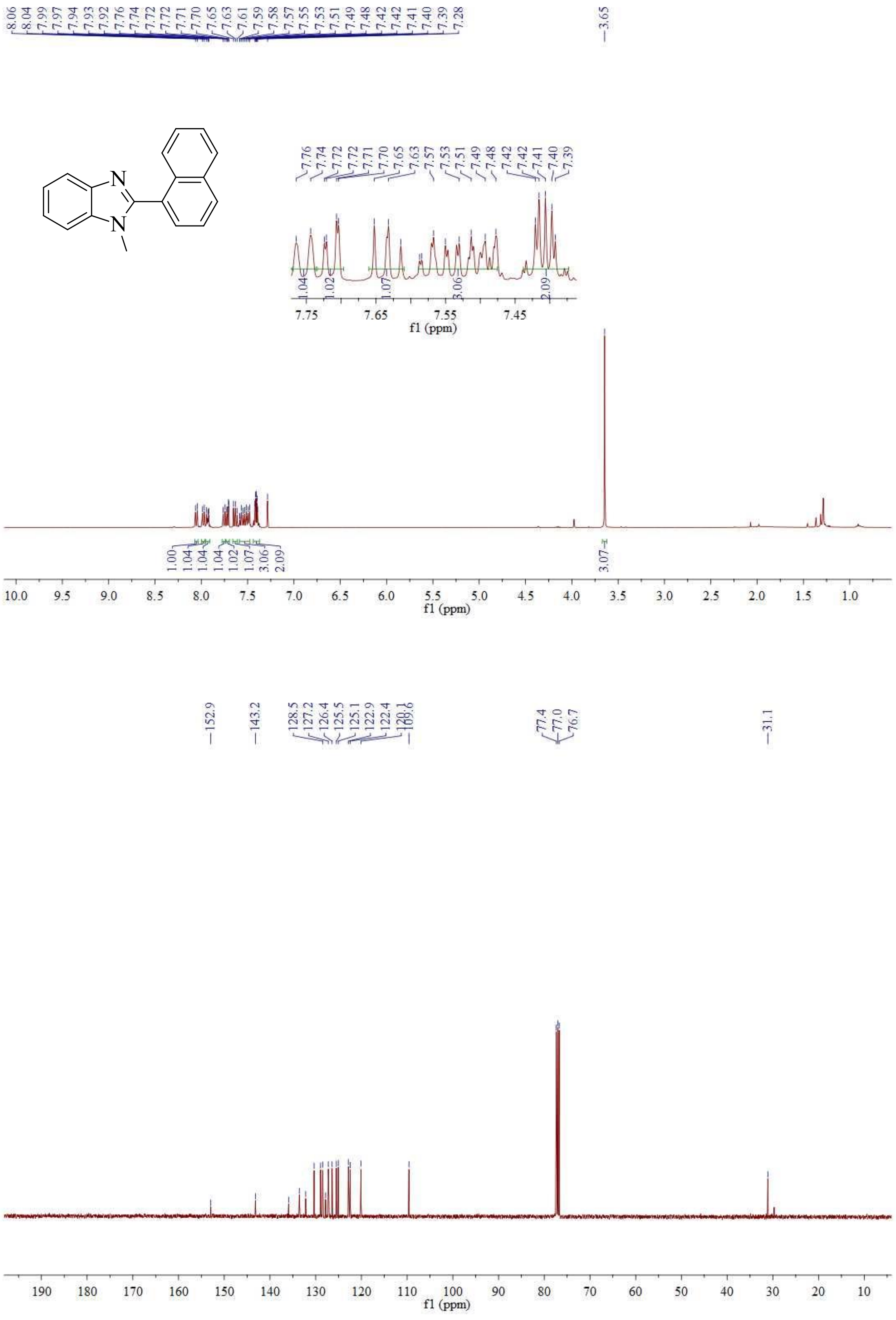

Fig. S22 ^{1}H (400 MHz) and ^{13}C {^{1}H} (100 MHz) NMR spectra of **3v** in $CDCl_3$
